# Synthesis of Mixed Arylalkyl Tertiary Phosphines via the Grignard Approach

**DOI:** 10.3390/molecules27134253

**Published:** 2022-07-01

**Authors:** Ashanul Haque, Khalaf M. Alenezi, Hani El Moll, Muhammad S. Khan, Wai-Yeung Wong

**Affiliations:** 1 Department of Chemistry, College of Science, University of Hail, Ha’il 81451, Saudi Arabia; k.alenezi@uoh.edu.sa (K.M.A.); h.elmoll@uoh.edu.sa (H.E.M.); 2 Department of Chemistry, Sultan Qaboos University, P.O. Box 36, Al-Khod 123, Oman; 3 Department of Applied Biology and Chemical Technology, The Hong Kong Polytechnic University, Hung Hom, Kowloon, Hong Kong, China

**Keywords:** Grignard reaction, cross-coupling, organophosphorus chemistry, phosphines, trivalent phosphorus

## Abstract

Trialkyl and triaryl phosphines are important classes of ligands in the field of catalysis and materials research. The wide usability of these low-valent phosphines has led to the design and development of new synthesis routes for a variety of phosphines. In the present work, we report the synthesis and characterization of some mixed arylalkyl tertiary phosphines via the Grignard approach. A new asymmetric phosphine is characterized extensively by multi-spectroscopic techniques. IR and UV–Vis spectra of some selected compounds are also compared and discussed. Density functional theory (DFT)-calculated results support the formation of the new compounds.

## 1. Introduction

Low-valent chemically functional phosphines or P(III) compounds are an important building block in organic, main group, and organometallic chemistry. They have found immense applications in the area of catalysis [1], as well as the development of new materials for theranostic [2,3] and opto-electronic (O-E) [4] applications. It has been demonstrated that via a minor modification in the phosphine core, it is possible to modulate the properties and applications of the resulting materials. We have a longstanding interest in the design and development of phosphine-coordinated transition-metal-containing metalla-ynes and poly(metalla-ynes). We and others have demonstrated that the nature of aryl phosphines not only influences the solubility of the material, but also helps to fine-tune its other properties [5,6]. Similarly, by late-stage sulfonation of the aryl phosphine, water-soluble symmetric and asymmetric phosphines can be produced for applications in aqueous organometallic chemistry and homogeneous catalysis [7]. Owing to this, synthesis of functionalized trialkyl and triaryl phosphines has proven to be an important topic in organic synthesis.

Among other methods, symmetric/asymmetric alkyl and aryl phosphines are mainly obtained via the cross-coupling reaction between an organometallic compound and halogenated phosphines or phosphanes under controlled conditions [8]. Moreover, synthesis and characterization of symmetric triarylphosphines through Grignard reagents has also been reported [9,10,11,12]. Thiel et al. [13] reported synthesis of triphenylphosphine ligands bearing pyrazole or 2-aminopyrimidine groups and their Pd(II) complexes using this protocol. Ragaini et al. [14] reported preparation of triarylphosphines with para –SH and –SMe groups by reacting Ar-MgBr with PCl_3_. Frisch and Lyons [15] reported synthesis of tris-(p-trimethylsilylphenyl)-phosphine via the reaction of PCl_3_ or PCl_5_ with a corresponding Grignard reagent.

Despite these advances, the development of efficient methods to construct asymmetric P-C remains a challenge. Therefore, application of milder methods using air-stable and economical starting materials would be most desirable. From the materials science point of view, it is desirable to construct new phosphines with balanced electronic interactions and steric properties. We report herein the synthesis and characterization of a series of mixed arylalkyl tertiary phosphines via the Grignard approach.

## 2. Results and Discussion

### 2.1. Synthesis

**Figure 1:**
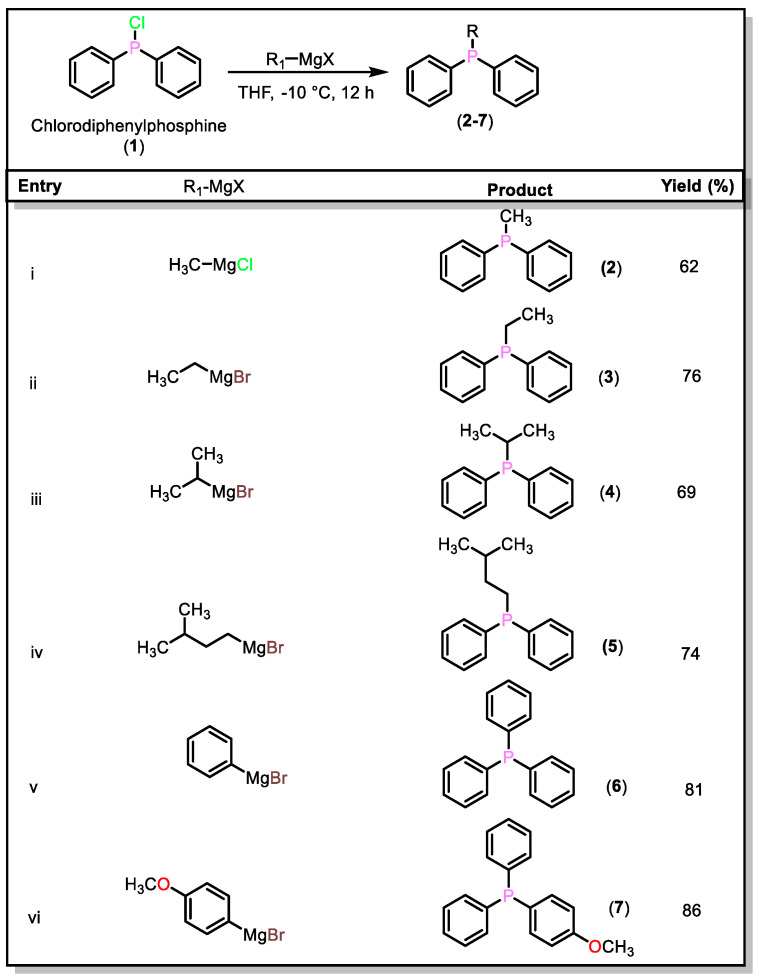
Synthesis of mixed arylalkyl and triaryl phosphines starting from chlorodiphenylphosphine.

The main objective of this work is to establish the utility of the Grignard approach for preparing new as well as previously reported available mixed arylalkyl tertiary phosphines. In this context, chlorodiphenylphosphine and dichlorophenylphosphine were coupled with different alkyl and aryl Grignard reagents (method A–C, Appendix A). For example, compound (**2**) was obtained by treating methyl magnesium chloride (CH_3_MgCl, 3.0 M solution in THF) with chlorodiphenylphosphine (Ph_2_PCl, **1**) in THF at −10 °C (method A, Appendix A). Other aliphatic (**3**–**5**, Figure 1) and aromatic (**6**–**7**, Figure 1) phosphines were obtained in a similar manner, using commercially available Grignard reagents with good yields (62–86%). It was noted that the synthesis of trisubstituted phosphines using aromatic Grignard reagents was better than that using aliphatic Grignard reagents under aerobic conditions; products in the latter case showed signs of oxidation reaction.

The molecular structure of the compounds was confirmed by FTIR, along with one- (^1^H, ^13^C, and ^31^P-NMR) and two-dimensional NMR spectroscopic techniques (vide infra and Figures S1–S11 in the Supplementary Materials). In the IR spectra, characteristic peaks for P-Alk at around 1450–1395 cm^−1^ (asym. def.) and 1346–1255 cm^−1^ (sym. def.), along with P-Ar stretching at around 1130–1090 cm^−1^, suggested the formation of the products. Moreover, the lack of strong peaks at around 1140–1320 cm^−1^ excluded the formation of oxidation products. Proton-decoupled ^31^P(^1^H) NMR spectra showed resonance between ~−7 ppm and −27 ppm (Supplementary Materials), which was significantly dependent upon the substituents (the more basic the phosphine, the more up-field the signal in the ^31^P NMR spectra). In the past, one- or multi-step synthesis of compounds (**2**) [16,17] (**3**) [18], (**4**) [19], (**5**) [20], (**7**) [21], (**9**) [22], (**10**) [23], and (**11**) [24] has been reported, including by the Grignard method, with which our results match well. Moreover, in our method, the yields were comparable to the transition-metal-mediated synthesis with equally broad scope. For instance, Jiang et al. [16] reported the synthesis of (**2**) with a 74% yield in a reaction conducted at −35 °C, while we found a yield of 66% at a moderate temperature. Compound (**5**) was reported using LiPPh_2_ and alkyl bromide, but no yield was mentioned. We also isolated compound (**7**) with a yield similar to that obtained in an earlier report [21]. Recently, some other researchers reported the synthesis of compound (**7**) using metal-catalyzed [25] or metal-free reaction [26] of 4-bromoanisole and diphenylphosphine.

**Figure 2:**
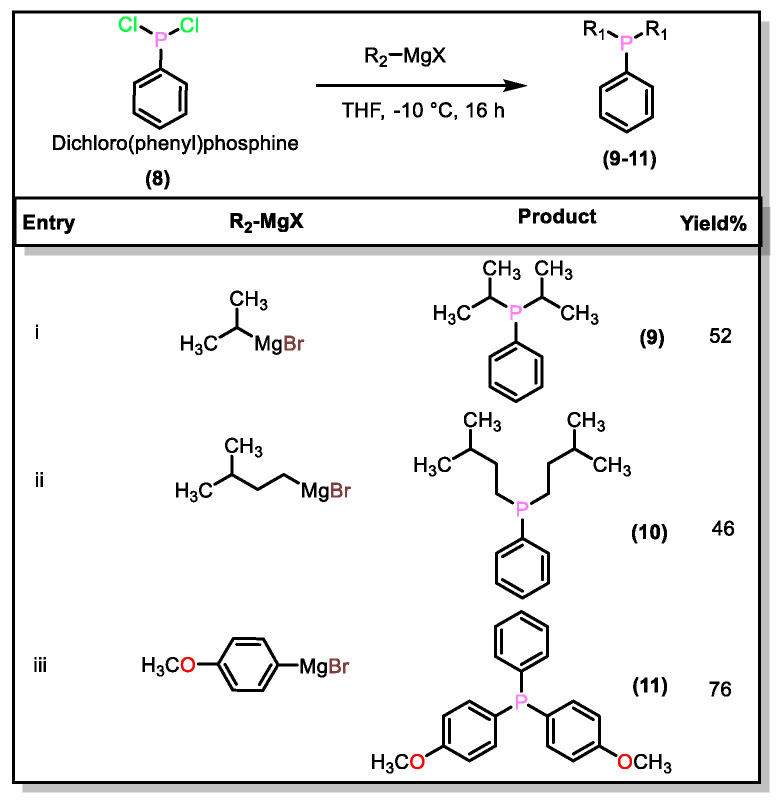
Synthesis of mixed arylalkyl and triaryl phosphines starting from dichlorophenylphosphine.

After successful investigation of the Grignard reaction on (**1**) (method A, Appendix A), we investigated the reaction of a Grignard reagent with dichlorophenylphosphine (PhPCl_2_, **8**) for the synthesis of asymmetric phosphine (method B, Appendix A). In this case, we used a slight excess of Grignard reagent to afford the mixed dialkylated and diarylated product. In a typical procedure, the addition of 2.5 equivalents of isopropylmagnesium bromide (*^i^*Pr-MgBr) to the solution of (**8**) in THF at −10 °C afforded diisopropylphenylphosphine (**9**) at a 52% yield. The other derivatives were prepared following a similar procedure, using 2.5 equivalents of the respective Grignard reagents with moderate yields (46–76%, Figure 2). For the aromatic phosphines (**11**, Figure 2), we used 2.5 equivalents of commercially available p-methoxyphenylmagnesium bromide (MeO-PhMgBr), and obtained the desired product (**11**) with a good yield. It is worth mentioning that the aryl Grignard reagents gave better conversion, as in the previous examples. However, when the alkyl Grignard reagents (R = Me, Et) were used, products in very low amounts with inseparable mixtures were obtained. Huang et al. [27] reported the synthesis of compound (**11**) via an in-situ-generated Grignard reagent at −78 °C. Compared to the reported method (yield = 52%), our method is less time-consuming and gives a higher yield (76%).

**Figure 3:**
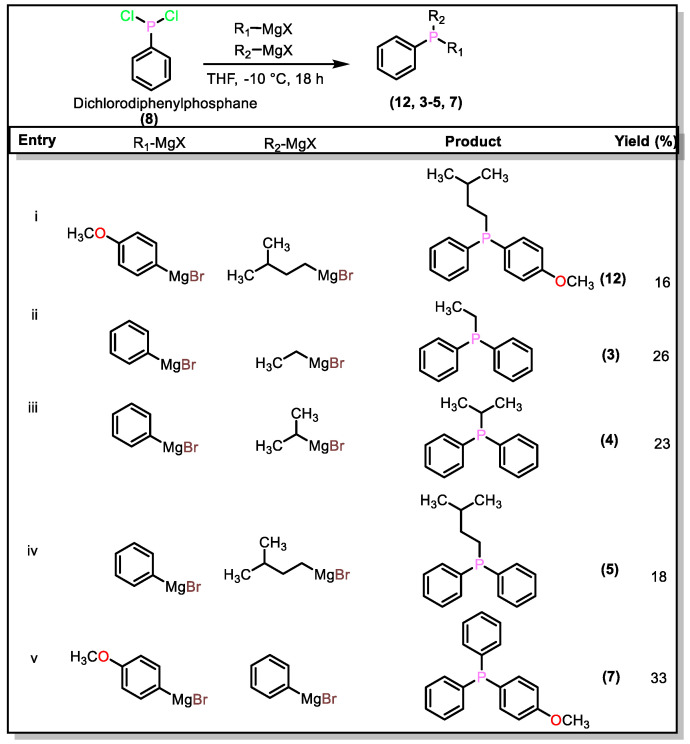
One-pot synthesis of mixed arylalkyl and triaryl phosphines.

Intrigued by the abovementioned results, attempts have also been made to obtain mixed phosphines under one-pot conditions (Figure 3). In a typical procedure, to a solution of (**8**) at −10 °C, one equivalent of a given R_1_-MgX was added, followed by the addition of one equivalent of another R_2_-MgX (method C, Appendix A). Upon controlled and slow addition of Grignard reagents, we noted the formation of asymmetric phosphines such as (**3–5**), (**7**), and (**12**), albeit with low yields (16–33%). Too fast or too slow addition of the reagents led to the formation of mixtures in greater amounts that were not separable. However, the reasons for the low yield could be manifold; we assume that factors such as competitive reaction between the different Grignard reagents, formation of cross-products, difficult separation (due to similar polarity of the products), etc., were mainly responsible. Currently, we are investigating and optimizing the conditions to develop a transition-metal-based catalyst-free protocol for the synthesis of asymmetric phosphines in one pot.

### 2.2. Structural Studies

#### 1D- and 2D-NMR Spectroscopy

In 2017, Kovács et al. [23] reported the production of compound (**10**) by the reduction of its oxide. However, the exact source and analytical data were not given, although there was nothing remarkable about its preparation. We conducted a full structural analysis of this compound, including multidimensional NMR spectroscopy. The ^1^H-NMR spectrum of compound (**10**) in CDCl_3_ shows 5 aromatic protons as a multiplet between δ 7.51–7.30 ppm, a multiplet at δ 1.74–1.62 ppm for 4 P-CH_2_ protons, one dpd at δ 1.58 ppm for 2 CH protons, δ 1.35–1.16 ppm for 4 P-CH_2_-CH_2_ protons, and one doublet at δ 0.87 ppm for 12 protons (Figure S8, Supplementary Materials). Compared to the other methylene group (i.e., H10 and H11), the chemical shift value of P-CH_2_ (i.e., H1 and H3) is clearly downfield, so the assignment is free of doubt. 

**Figure 4:**
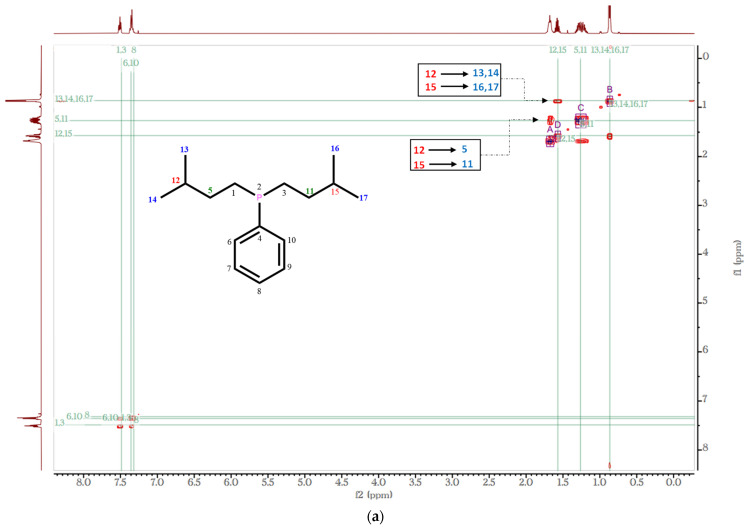

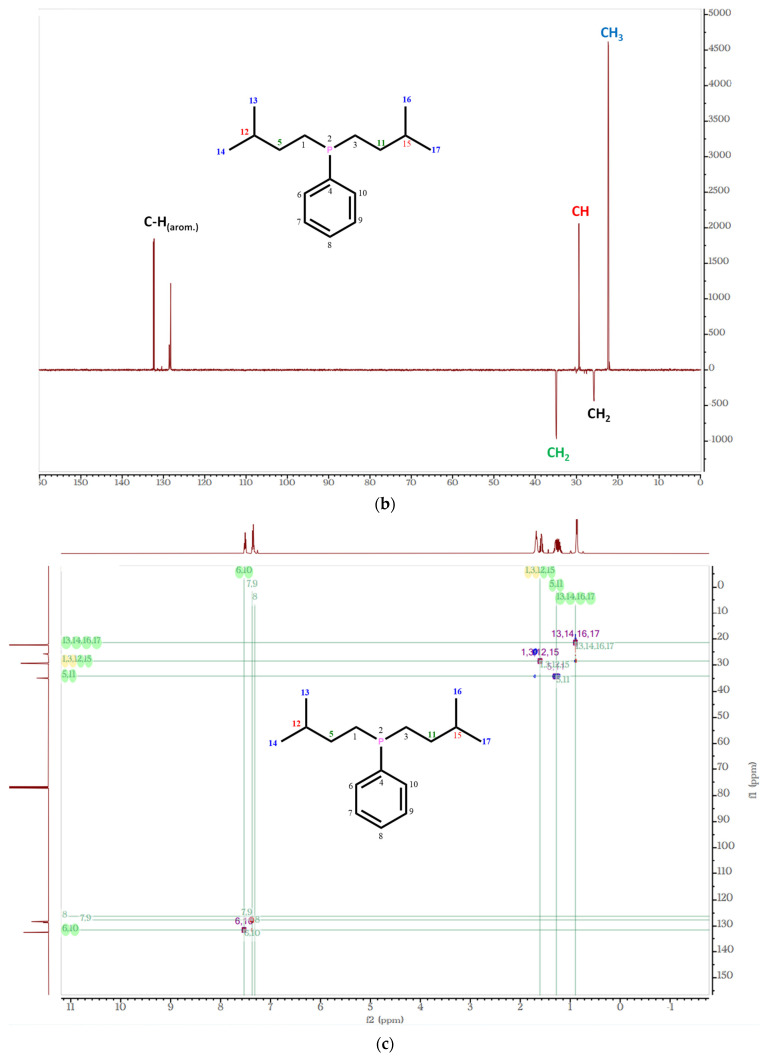
^1^H-^1^H COSY (**a**), DEPT-135 (**b**), and ^1^H-^13^C HSQC-COSY (**c**) spectra of compound (**10**).

The ^1^H-^1^H COSY results (Figure 4a) showed the expected coupling pattern: 12 equivalent protons attached to the methyl group (H13, H14, H16, and H17 at δ 0.87 ppm) coupling to methine protons (H12 and H15 at δ 1.58 ppm). The ^13^C NMR spectrum of the compound confirmed the structures assigned using ^1^H-NMR spectroscopic data. For instance, chemical shifts at δ 132.37–128.19 ppm (aromatic), δ 34.89–34.79 and δ 25.78–25.70 (methylene), δ 29.36–29.27 ppm (methine), and δ 22.28–22.20 ppm (methyl) were observed (Figure S8, Supplementary Materials). Note that the spectrum shows different shifts for aromatic carbons (C6/C10 and C7, C8, and C9). DEPT-135 (Figure 4b) clearly shows demarcation between the methylene (C5/C11 and C1/C3, negative peaks) and methine/methyl carbon (positive peaks). ^1^H-^13^C HSQC-COSY experiments (Figure 4c) were used to identify which hydrogen was attached to which carbon. For example, the ^13^C peak at ~δ 22 ppm is coupled to protons resonating at δ 0.87 ppm. Similarly, the peak at ~δ 25 ppm is coupled to up-field methine protons at δ 1.74–1.62 ppm. Further structural characterization was ascertained by HMBC (Figure S11, Supplementary Materials). This includes coupling of aromatic protons (δ 7.5–7.3 ppm) with carbon signals in the aromatic region.

**Figure 5:**
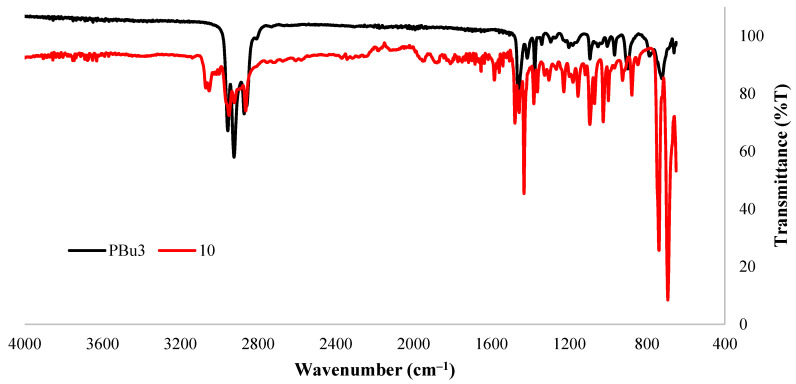
IR (ATR) spectra of compound (**10**) and tributylphosphine (PBu_3_).

Figure 5 depicts the IR spectra of compound (**10**) and a commercially available tributylphosphine (PBu_3_). Due to their structural similarity, the IR spectra were found to be overlapping in most of the region, except for those characteristics relating to aryl phosphines. The peak at 1433 cm^−1^ in (**10**) and 1457 cm^−1^ in PBu_3_ can be assigned to the deformation bands present in P-CH_2_-R-type phosphines. In addition, compound (**10**) shows P-Ar stretching at 1150 cm^−1^, aromatic C-H stretching at 3050 cm^−1^, and overtones which are absent in PBu_3_. 

**Figure 6:**
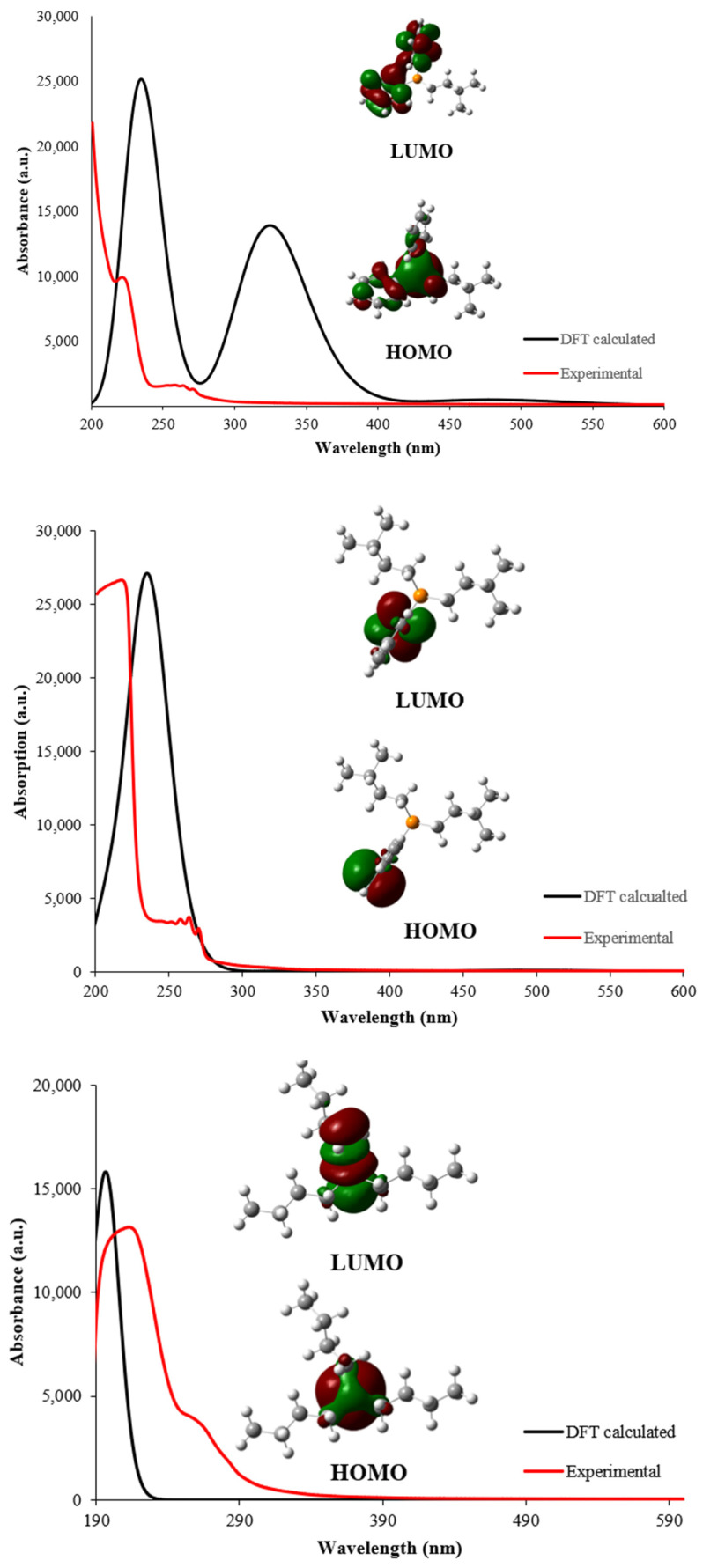
Theoretically calculated (black line) and experimental (red line) UV spectra of (**5**), (**10**), and tributylphosphine (PBu_3_) in acetonitrile at room temperature. Frontier molecular orbitals (HOMO/LUMO) are given in the inset.

The UV–Vis spectra of isopentyldiphenyl phosphine (**5**), diisopentyl(phenyl)phosphine (**10**), and a commercially available tributylphosphine (PBu_3_) were recorded in acetonitrile (Figure 6). Bands can be seen in the spectra in the expected positions. For instance, PBu_3_ exhibits high energy absorption maxima at 216 nm, while (**10**) shows maxima at 220 nm, along with a low-energy broad band in the region of ~260–270 nm with vibronic features. Similar, but slightly redshifted peaks can be seen in (**5**), owing to the presence of two phenyl groups. Note that (**5**) and (**10**) bear the features of the PBu_3_, methyldiphenyl phosphine, and triphenylphosphine [28]. While the high energy transition in PBu_3_ can be attributed to n → σ* transition, the absorption in the latter compounds (i.e., **5** and **10**) is due to n → π* transitions. This assignment is consistent with the theoretical calculations (vide infra) [29].

### 2.3. Density Functional Theory (DFT) Calculations

**Figure 7:**
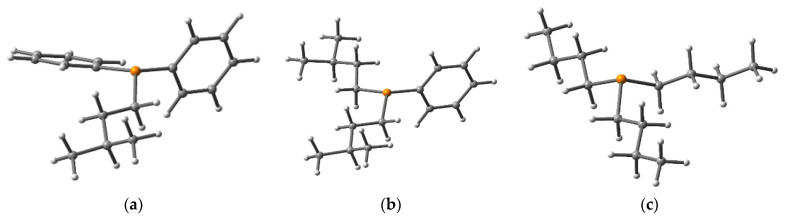
3D optimized structures of (**a**) compound (**5**), (**b**) compound (**10**), and (**c**) compound tributylphosphine (PBu_3_) from B3LYP calculations.

Density functional theory (DFT) is an important tool to underpin many structural features and photophysical processes [30,31,32,33,34,35]. Using this tool, one can determine the chemical stability, reactivity, etc., of any system [36]. The 3D optimized structures of compounds (**5**), (**10**), and tributylphosphine (PBu_3_), obtained by B3LYP calculations, are shown in Figure 7. As expected, all of the studied phosphines maintain the tetrahedral geometry around the phosphorus center. Even though (**5**) has one while (**10**) has two phenyl groups, no significant differences in the structure or the highest occupied molecular orbital (HOMO)–lowest unoccupied molecular orbital (LUMO) gap were noted (Figure 6, inset).

Overlaid experimentally and theoretically calculated UV spectra at the DFT level are depicted in Figure 6 (top), while the data are presented in Table 1. The topology of the HOMO and LUMO is depicted in Figure 7 (inset). According to the calculations, the most intense absorption corresponds to an n → π* transition in (**5**) and (**10**) and an n → σ* transition in PBu_3_, from HOMO to LUMO (see inset, Figure 7). For PBu_3_, both HOMO to LUMO and HOMO to LUMO + 1 transitions contribute to the UV spectrum. The molecular orbitals are delocalized over the aromatic parts (phenyl) of (**5**) and (**10**). HOMO–LUMO gaps for (**5**), (**10**), and PBu_3_ were found to be 3.5, 3.5, and 5.8 eV, respectively, consistent with previous works on phosphorus-based compounds [29,37]. 

## 3. Materials and Methods

### 3.1. General Procedures

All reactions were conducted under an inert atmosphere using standard Schlenk techniques. Unless stated otherwise, all chemicals were obtained from Sigma-Aldrich and used without further purification. NMR spectra were recorded on Bruker MM-250 and WM-400 spectrometers in CDCl_3_. The ^1^H and ^13^C NMR spectra were referenced to solvent resonances, and the ^31^P NMR spectra were referenced to an external phosphoric acid standard (85% H_3_PO_4_). Splitting patterns are designated as follows: s, singlet; d, doublet; t, triplet; q, quartet; m, multiplet. Chemical shift values are given in ppm. IR spectra were recorded using a Cary 630 FTIR spectrometer. Absorption spectra were recorded on a Varian Cary 50 UV–Visible spectrophotometer in a 1 cm quartz cuvette. Liquid chromatography/mass spectrometry (LC/MS) was performed on an Agilent LC/MS instrument (1260 Infinity II) equipped with a reverse-phase C_18_ column (2.7 μm particle size, 3.0 × 100 mm), electrospray (ESI) mass spectrometry detector, and photodiode array detector. The ground state of the phosphine compounds was calculated using the Gaussian 2009 program package [38]. The Becke–Lee–Young–Parr composite exchange correlation functional (B3LYP) [39,40] method with the 6-311G(d,p) [41] basis set was used for the geometric optimization and the energy level calculation. All optimized geometries were subjected to vibrational frequency analysis to ensure that they corresponded to local minima without imaginary frequencies. Natural bond orbital (NBO) analysis [42] was performed at the same level of theory. The electronic structure was examined in terms of the highest occupied molecular orbitals (HOMOs) and the lowest unoccupied molecular orbitals (LUMOs). TD-DFT calculations using the B3LYP functional and the 6-311G(d,p) basis set were used for the prediction of the UV spectra of the phosphine compounds. Cartesian coordinates for the optimized structures are presented in Tables S1–S3 (Supplementary Materials).

### 3.2. Synthesis and Characterization

*Method A*: To the stirred solution of chlorodiphenylphosphine (1.0 equiv.) in THF (15–20 mL), a commercially available molar solution of alkyl/aryl magnesium halide (1.1 equiv.) was added dropwise at −10 °C and stirred for 12 h. The reaction was quenched by adding half-saturated NH_4_Cl solution (prepared using deoxygenated water), diluted with ethyl acetate (10–20 mL), and stirred for 15 min. The organic layer was separated, and the aqueous layer was washed with ethyl acetate (3 × 15 mL). The combined organic layer was dried over anhydrous Na_2_SO_4_ and concentrated under vacuum. The crude product was purified by flash column chromatography.

*Method B*: To the stirred solution of dichlorophenylphosphine (1.0 equiv.) in THF (15 mL), a commercially available molar solution of alkyl/aryl magnesium halide (2.5 equiv.) was added dropwise at −10 °C and stirred for 12 h. The reaction was quenched by adding half-saturated NH_4_Cl solution (prepared using deoxygenated water), diluted with ethyl acetate (10–20 mL), and stirred for 15 min. The organic layer was separated, and the aqueous layer was washed with ethyl acetate (3 × 15 mL). The combined organic layer was dried over anhydrous Na_2_SO_4_ and concentrated under vacuum. The crude product was purified by flash column chromatography.

*Method C*: To the stirred solution of dichlorophenylphosphine (1.0 equiv.) in THF (10 mL) at −10 °C, alkyl/aryl magnesium halide (1.0 equiv.) was added dropwise, and the mixture was stirred for 6 h. Following this, a second Grignard reagent (1.0 equiv.) was added dropwise to the reaction mixture at the same temperature, and it was stirred overnight. The reaction was quenched by adding half-saturated NH_4_Cl solution (prepared using deoxygenated water), diluted with ethyl acetate (10–20 mL), and stirred for 15 min. The organic layer was separated, and the aqueous layer was washed with ethyl acetate (3 × 15 mL). The combined organic layer was dried over anhydrous Na_2_SO_4_ and concentrated under vacuum. The crude product was purified by flash column chromatography.

## 4. Conclusions

In conclusion, a variety of functionalized symmetric and asymmetric phosphines were prepared via the operationally simple phosphination of Grignard reagents in THF. However, attempts to prepare asymmetric phosphines under one-pot conditions were not very successful, and some other phosphines were obtained with yields better than those previously reported. Furthermore, we also performed extensive structural characterizations—such as multidimensional NMR—to establish the chemical structures of the products. DFT calculations also supported the spectral data of the studied compounds.

## Figures and Tables

**Table 1:** Absorption data and band gaps of (**5**), (**10**), and tributylphosphine (PBu_3_).

Compound	Absorption Maxima (nm)		Band Gap (Eg) ^1^
	Calc. ^1^	Exp. ^2^	
PBu_3_	199	216	5.8
(**10**)	238	220	3.5
(**5**)	238, 330	225, 263	3.5

^1^ Calculated at the DFT-B2LYP level. ^2^ Absorption spectra collected in acetonitrile at room temperature.

## Data Availability

The data presented in this study are available upon request from the corresponding author.

## Supplementary Materials

The following supporting information can be downloaded at: https://www.mdpi.com/article/10.3390/molecules27134253/s1, **Figure S1**: ^1^H-NMR, ^13^C-NMR, and ^31^P-NMR spectra of (**2**) obtained by method A; **Figure S2**: ^1^H-NMR, ^13^C-NMR, and ^31^P-NMR spectra of (**3**) obtained by method A; **Figure S3**: ^1^H-NMR, ^13^C-NMR, and ^31^P-NMR spectra of (**4**) obtained by method A; **Figure S4**: ^1^H-NMR, ^13^C-NMR, and ^31^P-NMR spectra of (**5**) obtained by method A; **Figure S5**: ^1^H-NMR, ^13^C-NMR, and ^31^P-NMR spectra of (**6**) obtained by method A; **Figure S6**: ^1^H-NMR, ^13^C-NMR, and ^31^P-NMR spectra of (**7**) obtained by method A; **Figure S7**: ^1^H-NMR, ^13^C-NMR, and ^31^P-NMR spectra of (**9**) obtained by method B; **Figure S8**: ^1^H-NMR, ^13^C-NMR, and ^31^P-NMR spectra of (**10**) obtained by method B; **Figure S9**: ^1^H-NMR and ^13^C-NMR spectra of (**11**) obtained by method B; **Figure S10**: ^1^H-NMR, ^13^C-NMR, and ^31^P spectra of (**12**) obtained by method C; **Figure S11**: HMBC spectrum of compound (**10**) obtained by method B; **Table S1:** Cartesian coordinates for the optimized structure of (**5**); **Table S2:** Cartesian coordinates for the optimized structure of (10); **Table S3**: Cartesian coordinates for the optimized structure of PBu_3_.

## Sample Availability

Samples of the compounds **2**–**7** and **9**–**12** are available from the authors.

## Appendix A

**Experimental**

*Methyldiphenylphosphine* (**2**)

Chlorodiphenylphosphine (800 mg, 3.626 mmol) and methylmagnesium chloride (1.6 mL, 4.714 mmol, 3 M solution in THF) were reacted following method A to yield product (**2**) as a colorless liquid (450 mg, 62%). ^1^H NMR (500 MHz, CDCl_3_) δ 7.43 (ddt, J = 7.5, 5.5, 1.7 Hz, 4H), 7.38–7.30 (m, 6H), 1.65 (d, J = 3.3 Hz, 3H), ^13^C NMR (176 MHz, CDCl_3_): 139.9, 132.1, 130.4, 128.4, 12.4. ^31^P NMR (162 MHz, CDCl_3_) *δ* ppm: −26,78 ppm.

*Ethyldiphenylphosphine* (**3**)

Chlorodiphenyl phosphine (1.0 g, 4.532 mmol) and ethylmagnesium bromide (4.9 mL, 5.892 mmol, 1.0 M solution in THF) were reacted following method A to yield product (**3**) as a colorless liquid (739 mg, 76%). ^1^H NMR (500 MHz, CDCl_3_) δ 7.48–7.41 (m, 4H), 7.39–7.29 (m, 6H), 2.08 (q, J = 7.6 Hz, 2H), 1.11 (dt, J = 17.0, 7.6 Hz, 3H). ^13^C NMR (176 MHz, CDCl_3_): 138.4, 132.6, 128.4, 20.5, 9.9. ^31^P NMR (162 MHz, CDCl_3_) *δ* ppm: −11.46 ppm.

*Isopropyldiphenylphosphine* (**4**)

Chlorodiphenylphosphine (1.0 g, 4.532 mmol) and ethylmagnesium bromide (5.9 mL, 5.892 mmol, 1 M solution in THF) were reacted following method A to yield product (**4**) as a colorless liquid (715 mg, 69%). ^1^H NMR (500 MHz, CDCl_3_) δ 7.47–7.38 (m, 4H), 7.30–7.20 (m, 6H), 2.43–2.32 (m, 1H), 1.00 (dd, J = 15.5, 6.9 Hz, 6H). ^13^C NMR (176 MHz, CDCl_3_): 138.4, 132.6, 128.4, 20.5, 9.9. ^31^P NMR (162 MHz, CDCl_3_) *δ* ppm: −12.50 ppm.

*Isopentyldiphenylphosphine* (**5**)

Chlorodiphenylphosphine (700 mg, 3.181 mmol) and isopentylmagnesium bromide (2.1 mL, 4.136 mmol, 2 M solution in ether) were reacted following method A to yield product (**5**) as a colorless liquid (602 mg, 74%). ^1^H NMR (500 MHz, CDCl_3_) δ 7.44 (tt, J = 7.3, 1.9 Hz, 4H), 7.37–7.28 (m, 6H), 2.12–2.00 (m, 2H), 1.66 (dpd, J = 13.3, 6.6, 1.0 Hz, 1H), 1.34 (dddd, J = 13.1, 11.7, 6.2, 3.3 Hz, 2H), 0.90 (d, J = 6.6 Hz, 6H). ^13^C NMR (176 MHz, CDCl_3_): 138.4, 132.6, 128.4, 34,7, 29.2, 25.5, 22.2. ^31^P NMR (162 MHz, CDCl_3_) *δ* ppm: −15.46 ppm.

*Triphenylphosphine* (**6**)

Chlorodiphenylphosphine (800 mg, 3.626 mmol) and phenylmagnesium bromide (4.7 mL, 4.714 mmol, 1 M solution in THF) were reacted following method A to yield product (**6**) as a white solid (771 mg, 81%). ^1^H NMR (500 MHz, CDCl_3_) δ 7.36 (m, J = 5.5, 3.2, 2.6 Hz, 15H). ^13^C NMR (176 MHz, CDCl_3_): 136.9, 133.8, 133.7, 128.5, 128.8. ^31^P NMR (162 MHz, CDCl_3_) *δ* ppm: −7.02 ppm.

*(4-Methoxyphenyl)diphenylphosphine* (**7**)

Chlorodiphenylphosphine (600 mg, 2.719 mmol) and 4-methoxy-phenylmagnesium bromide (3.5 mL, 3.535 mmol, 1 M solution in THF) were reacted following method A to yield product (**7**) as a white solid (684 mg, 86%). ^1^H NMR (500 MHz, CDCl_3_) δ 7.34–7.25 (m, 12 H), 6.91–6.85 (m, 2H), 3.79 (s, 3H). ^13^C NMR (176 MHz, CDCl_3_): 160.4, 137.4, 133.3, 135.5, 127.1, 128.4, 114.2, 55.1. ^31^P NMR (162 MHz, CDCl_3_) *δ* ppm: −8.6 ppm.

*Diisopropyl(phenyl) phosphine* (**9**)

Dichlorophenylphosphine (400 mg, 2.235 mmol) and isopropylmagnesium bromide (5.6 mL, 5.587 mmol, 1 M solution in THF) were reacted following method B to yield product (**9**) as a colorless liquid (200 mg, 46%). ^1^H NMR (500 MHz, CDCl_3_) δ 7.53–7.43 (m, 2H), 7.38–7.30 (m, 3H), 1.88–1.80 (m, 2H), 1.09 (dd, *J* = 15.2, 7.0 Hz, 6H), 0.92 (dd, *J* = 11.4, 6.9 Hz, 6H). ^13^C NMR (126 MHz, CDCl_3_) δ 134.59, 134.44, 131.51, 128.84, 128.20, 127.77, 67.94, 25.58, 22.59, 19.70, 18.62, 15.96. Note: additional peaks (around δ 4 ppm) were possibly due to the formation of oxides. Moreover, flash chromatography seemed to be ineffective in purifying the compounds; therefore, additional peaks in NMR could be seen.

*Diisopentyl(phenyl) phosphine* (**10**)

Dichlorophenylphosphine (1 g, 5.58 mmol) and isopentylmagnesium bromide (7 mL, 13.97 mmol, 2 M solution in ether) were reacted following method B to yield product (**10**) as a colorless liquid (287 mg, 46%). ^1^H NMR (500 MHz, CDCl_3_) δ 7.51 (ddd, J = 8.5, 5.1, 1.7 Hz, 2H), 7.38–7.30 (m, 3H), 1.74–1.62 (m, 4H), 1.58 (dpd, J = 13.2, 6.7, 0.9 Hz, 2H), 1.35–1.16 (m, 4H), 0.87 (d, J = 6.7 Hz, 12H). ^13^C NMR (126 MHz, CDCl_3_) δ 132.37, 132.22, 128.55, 128.25, 128.19, 34.89, 34.79, 29.36, 29.27, 25.78, 25.70, 22.28, 22.20. ^31^P NMR (162 MHz, CDCl_3_) *δ* ppm: −22.25 ppm.

*Bis(4-methoxyphenyl)(phenyl)phosphine* (**11**)

Dichlorophenylphosphine (500 mg, 2.794 mmol) and 4-methoxy-phenylmagnesium bromide (7.1 mL, 6.984 mmol, 1 M solution in THF) were reacted following method B to yield product (**12**) as a colorless liquid (687 mg, 76%). ^1^H NMR (500 MHz, CDCl_3_) δ 7.32–7.20 (m, 9H), 6.86 (dq, *J* = 7.6, 0.9 Hz, 4H), 3.78 (d, *J* = 0.9 Hz, 6H). ^13^C-NMR (126 MHz, CDCl_3_) δ 160.24, 138.30, 135.17, 132.99, 128.31, 128.28, 127.99, 114.14, 55.14.

*Isopentyl(4-methoxyphenyl)(phenyl) phosphine* (**12**)

Dichlorophenylphosphine (250 mg, 1.397 mmol), 4-methoxy-phenylmagnessiumbromide (1.4 mL, 1.397 mmol, 1 M solution in THF), and isopentylmagnesium bromide (0.8 mL, 1.397 mmol, 2 M solution in ether) were reacted following method C to yield product (**12**) as a colorless liquid. (64 mg, 16%). ^1^H NMR (500 MHz, CDCl_3_) δ 7.43–7.36 (m, 4H), 7.35–7.27 (m, 3H), 6.93–6.86 (m, 2H), 3.81 (s, 3H), 2.07–1.95 (m, 2H), 1.65 (dpd, J = 13.2, 6.7, 1.0 Hz, 1H), 1.41–1.22 (m, 2H), 0.90 (dd, J = 6.6, 1.4 Hz, 6H), ^13^C NMR (126 MHz, CDCl_3_) δ 160.29, 139.97, 139.87, 134.73, 134.57, 132.20, 132.06, 128.33, 128.29, 128.11, 55.18, 34.87, 29.43, 26.01, 22.27. ^31^P NMR (162 MHz, CDCl_3_) *δ* ppm: −7.55 ppm.
