# Supplementary material for: Synthesis of Mixed Arylalkyl Tertiary Phosphines via the Grignard Approach
Source: Molecules. 2022 Jul 1;27(13):4253. doi: 10.3390/molecules27134253 (PMC9268331; doi:10.3390/molecules27134253)
Supplement: Supplementary file 1 [file molecules-27-04253-s001.zip › molecules-1775170-supplementary.pdf]

Synthesis of Mixed Arylalkyl Tertiary Phosphines via the Grignard Approach

Ashanul Haque [1,*], Khalaf M. Alenezi [1], Hani El Moll [1], Muhammad S. Khan [2,*] and Wai-Yeung Wong [3,*]

[1] Department of Chemistry, College of Science, University of Hail, Ha'il 81451, Saudi Arabia;

[2] Department of Chemistry, Sultan Qaboos University, P.O. Box 36, Al-Khod 123, Oman

[3] Department of Applied Biology and Chemical Technology, the Hong Kong Polytechnic University, Hung Hom, Kowloon, Hong Kong, China

* Correspondence: a.haque@uoh.edu.sa (A.H.); msk@squ.edu.om (M.S.K.); wai-yeung.wong@polyu.edu.hk (W.-Y.W.)

Supporting Information

Experimental

*Ethyldiphenylphosphine (**3**)*

Dichlorophenylphosphine (250 mg, 1.397 mmol), phenylmagnesium bromide (1.4 mL, 1.397 mmol, 1 M solution in THF) and ethylmagnesium bromide (1.4 mL, 1.397 mmol, 1 M solution in THF) were reacted by following method C to yield product (**3**) as a colorless liquid (78 mg, 26 %). ^{1}H NMR (500 MHz, $CDCl_3$) δ 7.48 – 7.38 (m, 4H), 7.38 – 7.28 (m, 6H), 2.06 (q, J = 7.6 Hz, 2H), 1.10 (dt, J = 17.1, 7.6 Hz, 3H), ^{13}C NMR (126 MHz, $CDCl_3$) δ 138.82 (d, J = 2.1 Hz), 138.72, 132.82, 132.67, 128.49, 128.42, 128.36, 20.66 (d, J = 10.2 Hz), 10.13 (d, J = 16.4 Hz)

*Isopropyldiphenylphosphine (**4**)*

Dichlorophenyl phosphine (200 mg, 1.117 mmol), phenylmagnesium bromide (2.2 mL, 1.117 mmol, 1 M solution in THF) and isopropylmagnesium chloride (2.2 mL, 1.117 mmol, 1 M solution in THF) were reacted following method C to yield product as a colorless liquid (58 mg, 23 %). ^{1}H NMR (500 MHz, $CDCl_3$) δ 7.53 (dddd, J = 9.6, 6.0, 3.0, 1.3 Hz, 4H), 7.37 – 7.31 (m, 6H), 2.55 – 2.46 (m, 1H), 1.11 (d, J = 6.9 Hz, 3H), 1.08 (d, J = 6.9 Hz, 3H), 13C NMR (126 MHz, CDCl3) δ 133.65, 133.50, 131.54 (d, J = 2.7 Hz), 131.11, 131.04, 128.63, 128.54, 128.46, 128.40, 24.88, 19.39, 15.28

*Isopentyldiphenylphosphine (**5**)*

Dichlorophenylphosphine (300 mg, 1.676 mmol) in THF (10 mL), phenylmagnesium bromide (1.7 mL, 1.676 mmol, 2 M solution in THF) and isopentylmagnesium bromide (0.85 mL, 1.676 mmol, 1 M solution in ether) were reacted by following method C to yield product (**5**) as a colorless liquid (78 mg, 18 %). ^{1}H NMR (500 MHz, $CDCl_3$) δ 7.44 (ddq, J = 8.6, 6.3, 2.3 Hz, 4H), 7.39 – 7.29 (m, 6H), 2.09 – 2.02 (m, 2H), 1.67 (dtd, J = 13.2, 6.6, 0.9 Hz, 1H), 1.41 – 1.29 (m, 2H), 0.91 (d, J = 6.7 Hz, 6H), ^{13}C NMR (126 MHz, CDCl3) δ 138.91, 138.81, 132.74, 132.60, 128.43, 128.36, 128.31, 34.80, 29.38, 25.75, 22.24.

(4-Methoxyphenyl)diphenylphosphine (7)

Dichlorophenyl phosphine (200 mg, 1.117 mmol), phenylmagnesium bromide (1.1 mL, 1.117 mmol, 1 M solution in THF) and 4-methoxyphenylmagnesium bromide (1.05 mL, 1.117 mmol, 1 M solution in THF) were reacted by following method B to yield product (**7**) as colorless liquid (108 mg, 33 %). ^{1}H NMR (500 MHz, $CDCl_3$) δ 7.33 – 7.23 (m, 13H), 6.91 – 6.86 (m, 2H), 3.80 (s, 3H), ^{13}C NMR (126 MHz, $CDCl_3$) δ 160.36, 137.91, 137.83, 135.68, 135.51, 133.48, 133.32, 128.46, 128.43, 128.37, 127.63, 127.56, 114.26, 114.19, 55.17.

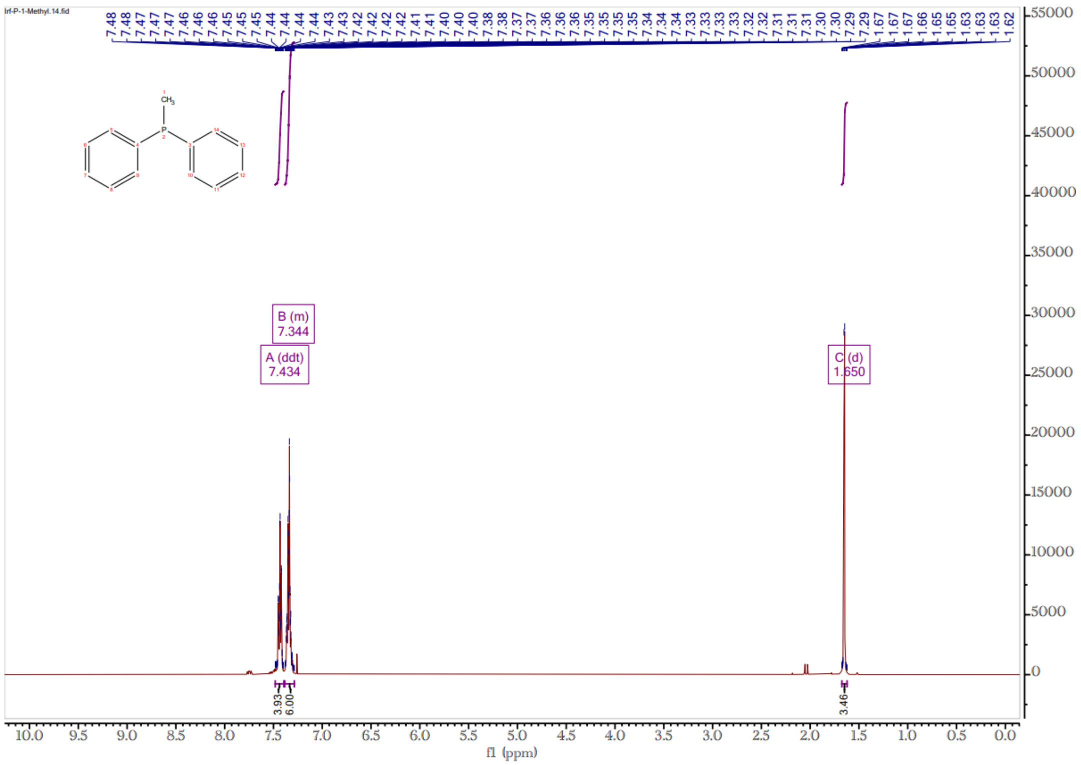

lrf-P-1-Methyl.14.fid
7.48 7.48 7.47 7.47 7.47 7.46 7.46 7.46 7.45 7.45 7.45 7.44 7.44 7.44 7.43 7.43 7.42 7.42 7.42 7.42 7.41 7.41 7.40 7.40 7.40 7.38 7.38 7.37 7.37 7.36 7.36 7.36 7.35 7.35 7.35 7.35 7.34 7.34 7.34 7.33 7.33 7.33 7.33 7.32 7.32 7.31 7.31 7.31 7.30 7.30 7.29 7.29 1.67 1.67 1.67 1.66 1.65 1.65 1.63 1.63 1.63 1.62
B (m)
7.344
A (ddt)
7.434
C (d)
1.650
3.93
6.00
3.46
f1 (ppm)

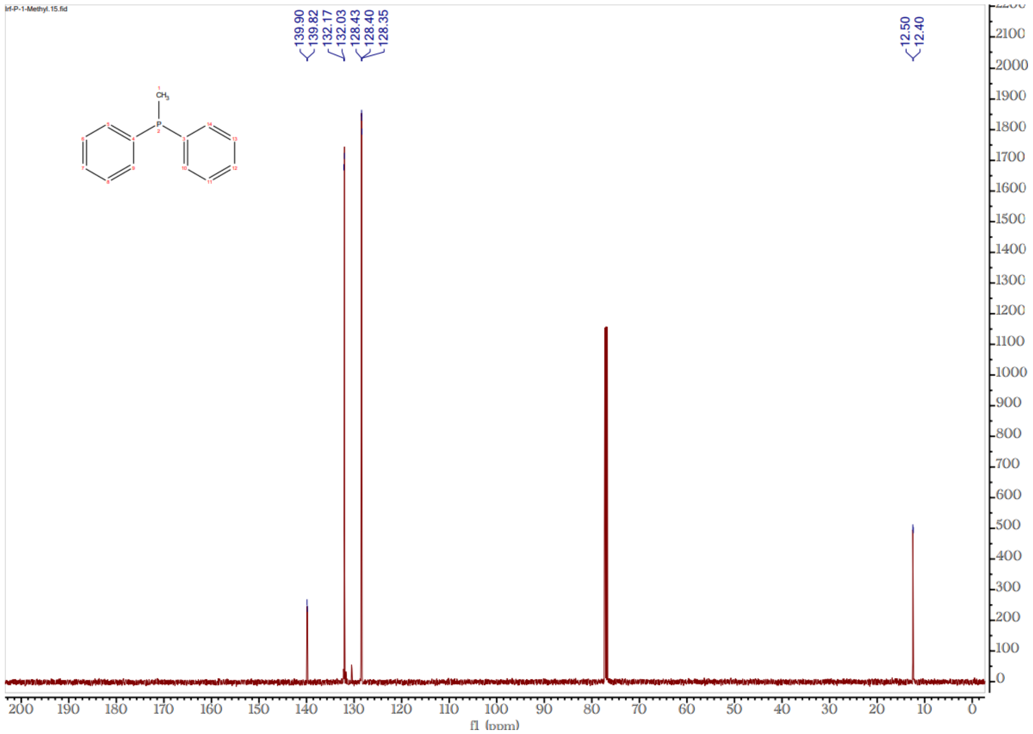

lrf-P-1-Methyl.15.fid
139.90
139.82
132.17
132.03
128.43
128.40
128.35
12.50
12.40
f1 (ppm)

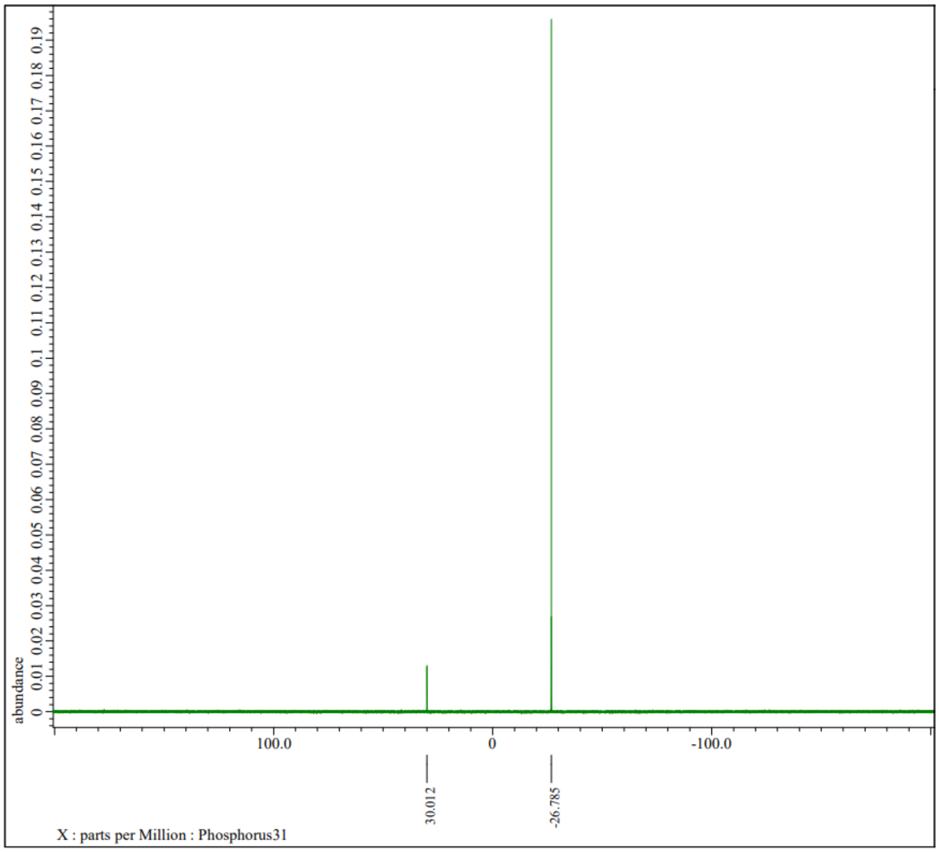

Figure S1. ¹H-NMR, ¹³C-NMR AND ³¹P-NMR spectra of (**2**) obtained by method A.

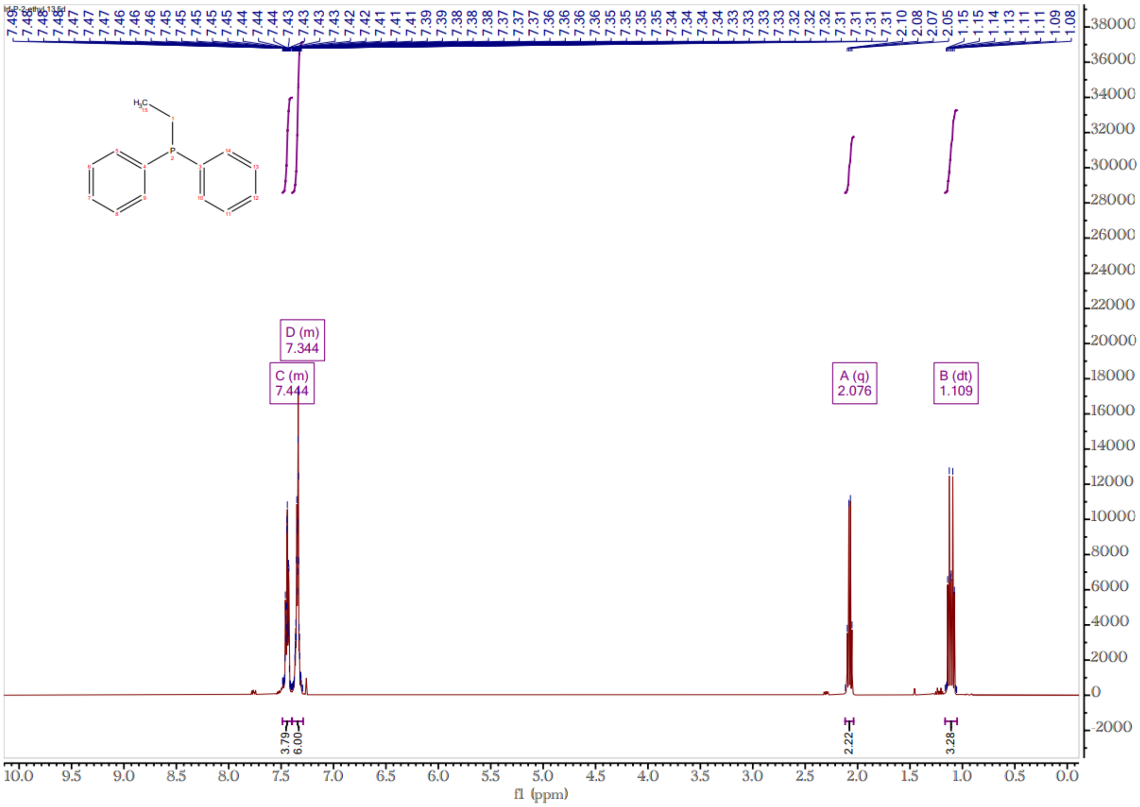

lrf-P-2-ethyl.13.fid
D (m)
7.344
C (m)
7.444
A (q)
2.076
B (dt)
1.109
3.79
6.00
2.22
3.28
f1 (ppm)

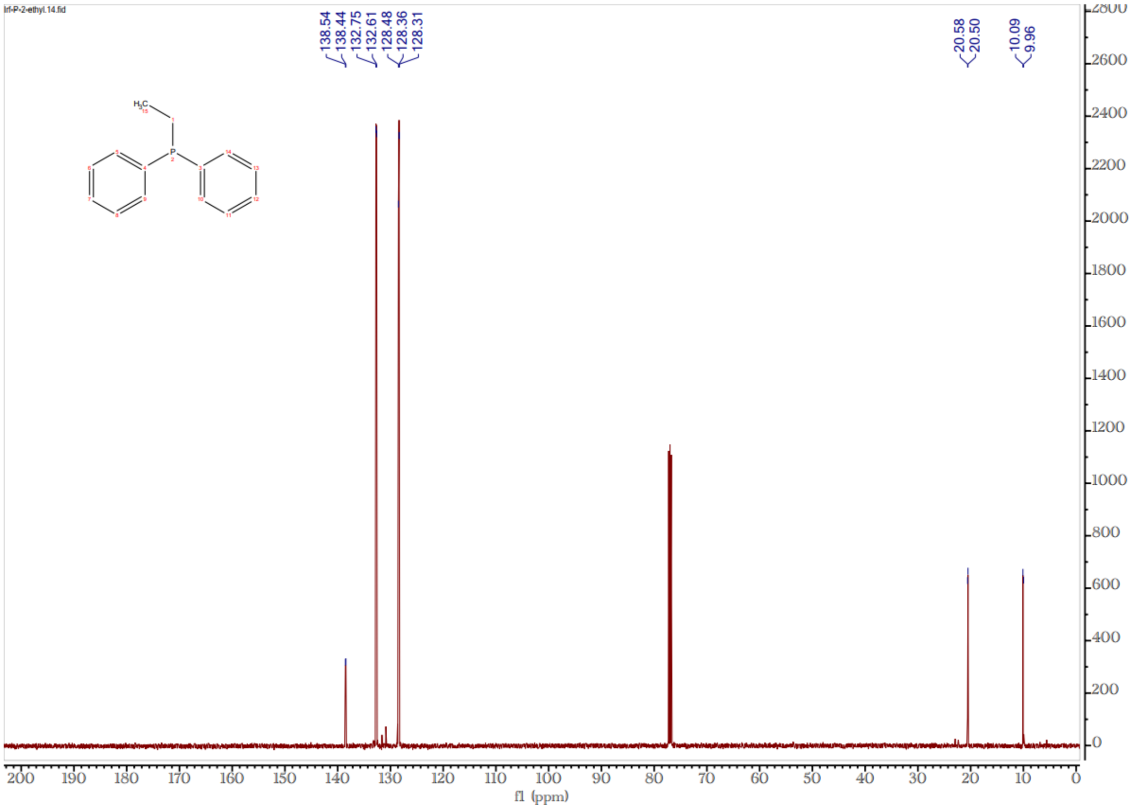

lrf-P-2-ethyl.14.fid
138.54
138.44
132.75
132.61
128.48
128.36
128.31
20.58
20.50
10.09
9.96
f1 (ppm)

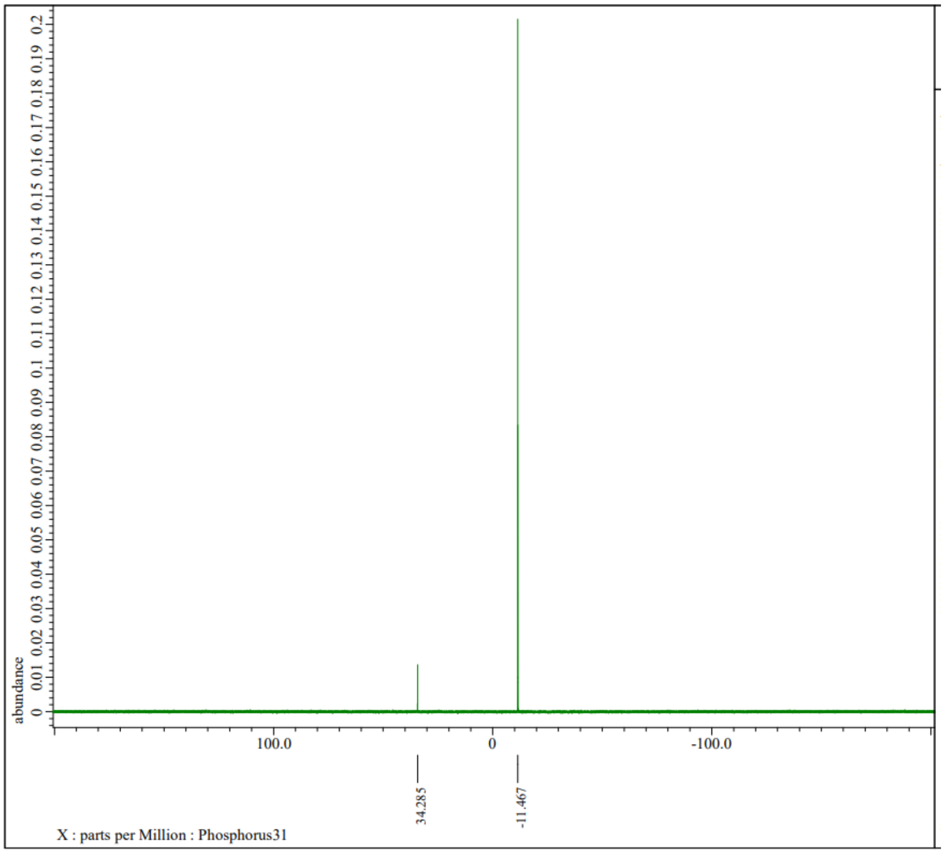

Figure S2. ^{1}H-NMR, ^{13}C-NMR and ^{31}P-NMR spectra of **(3)** obtained by method A.

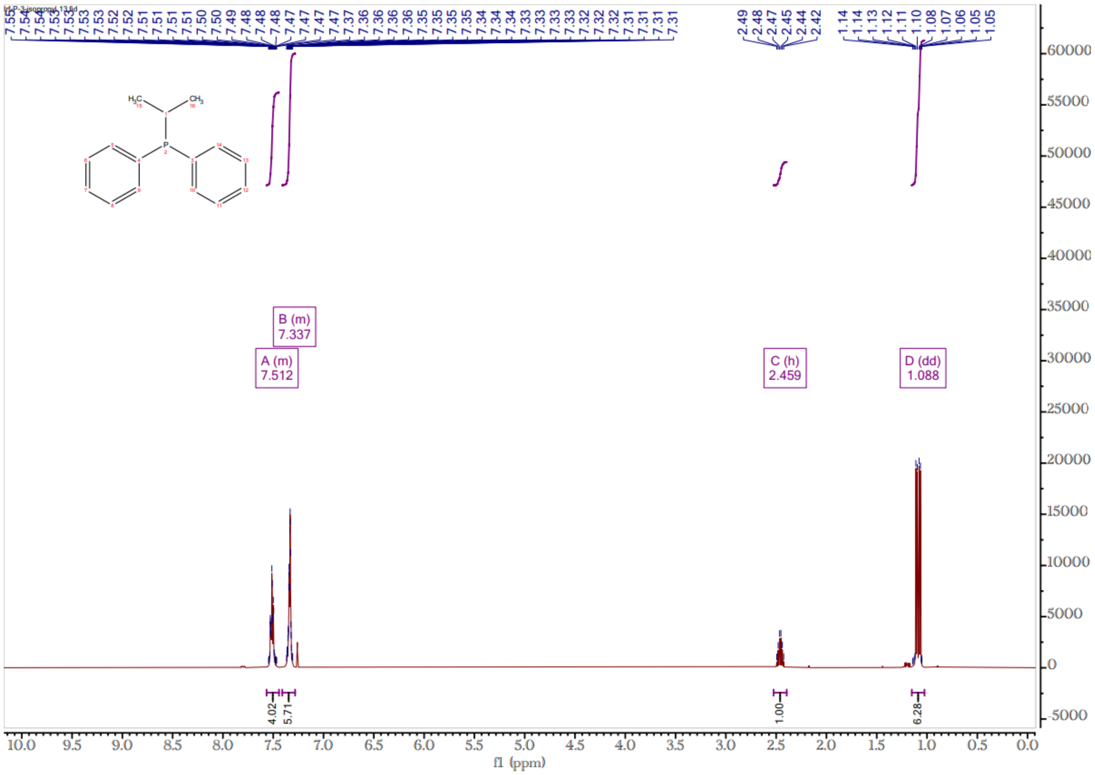
lrf-P-3-isopropyl.13.fid
A (m)
7.512
B (m)
7.337
C (h)
2.459
D (dd)
1.088
4.02
5.71
1.00
6.28
f1 (ppm)

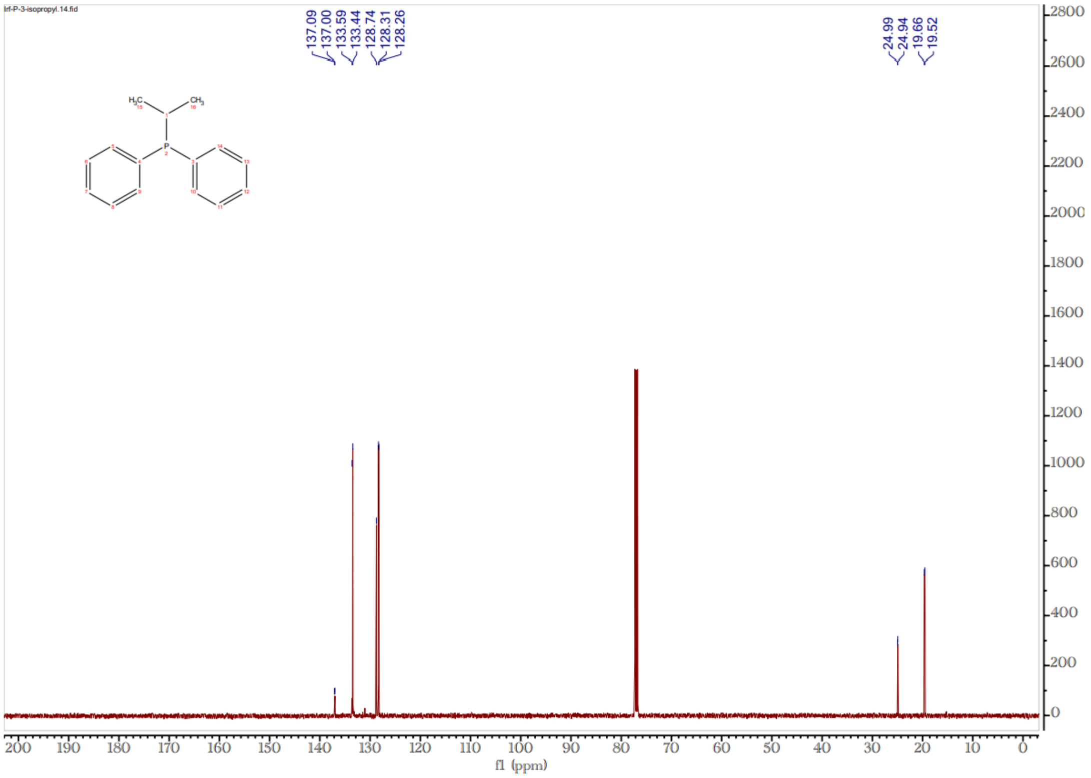
lrf-P-3-isopropyl.14.fid
137.09
137.00
133.59
133.44
128.74
128.31
128.26
24.99
24.94
19.66
19.52
f1 (ppm)

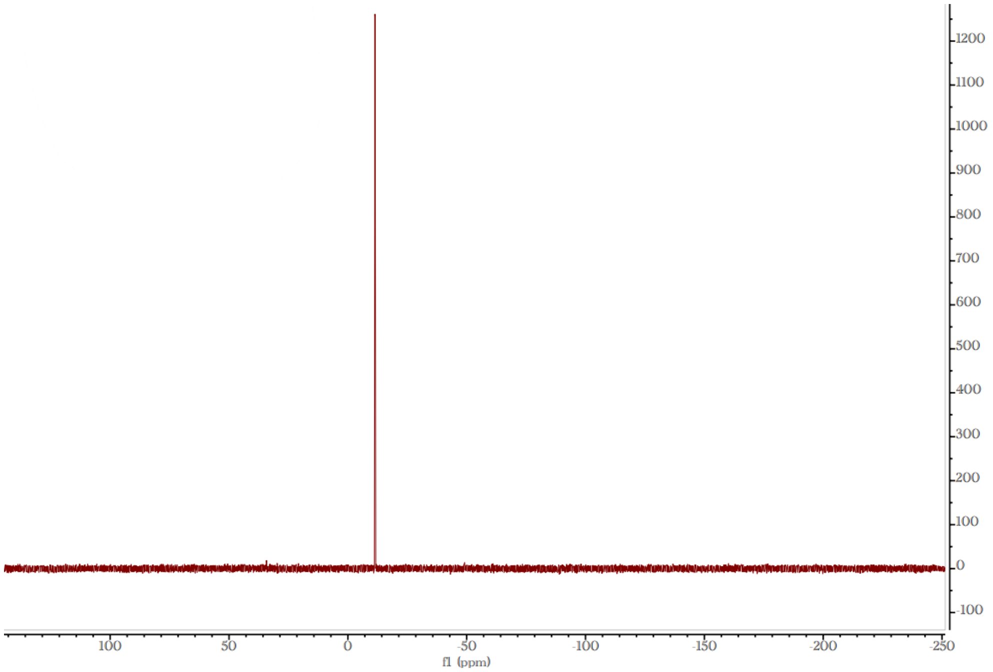

Figure S3. ^{1}H-NMR, ^{13}C-NMR and ^{31}P-NMR spectra of **(4)** obtained by method A.

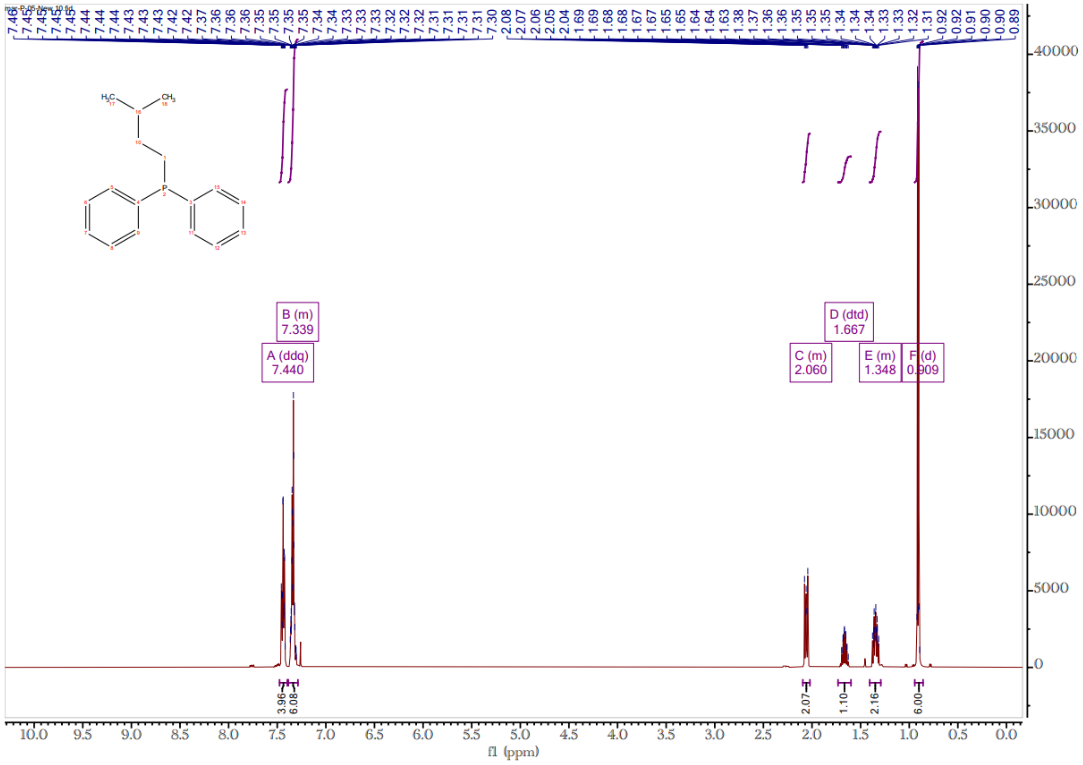

A (ddq)
7.440
B (m)
7.339
C (m)
2.060
D (dtd)
1.667
E (m)
1.348
F (d)
0.909
f1 (ppm)

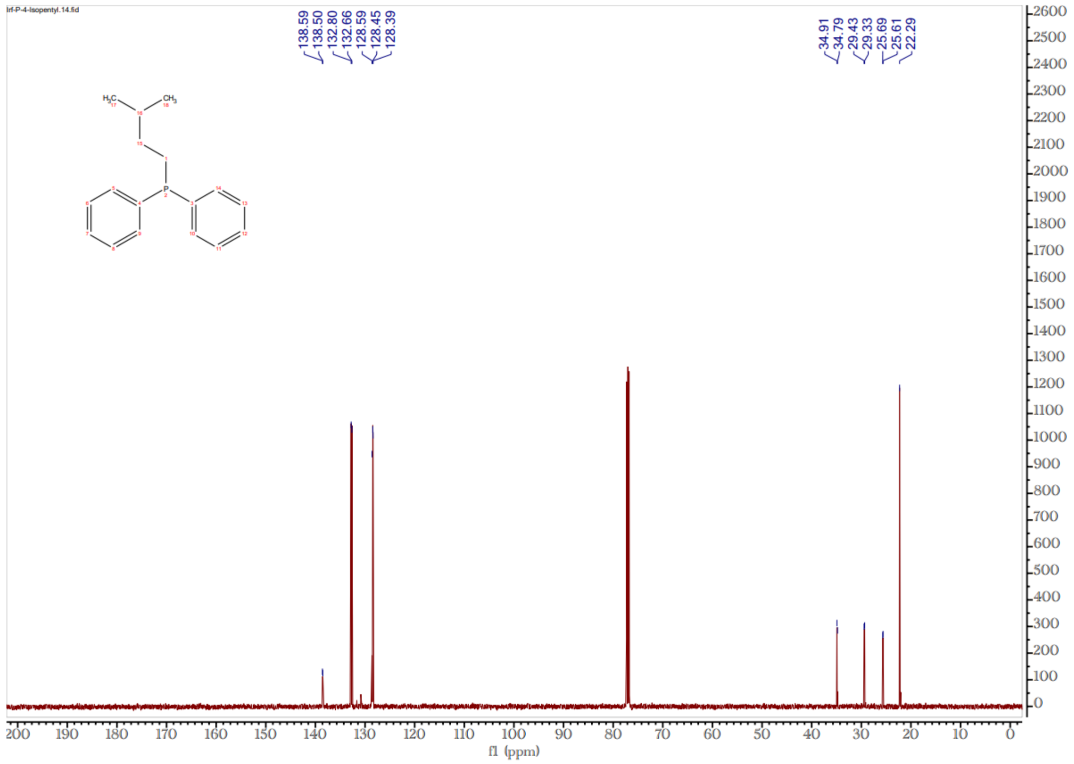

138.59
138.50
132.80
132.66
128.59
128.45
128.39
34.91
34.79
29.43
29.33
25.69
25.61
22.29
f1 (ppm)

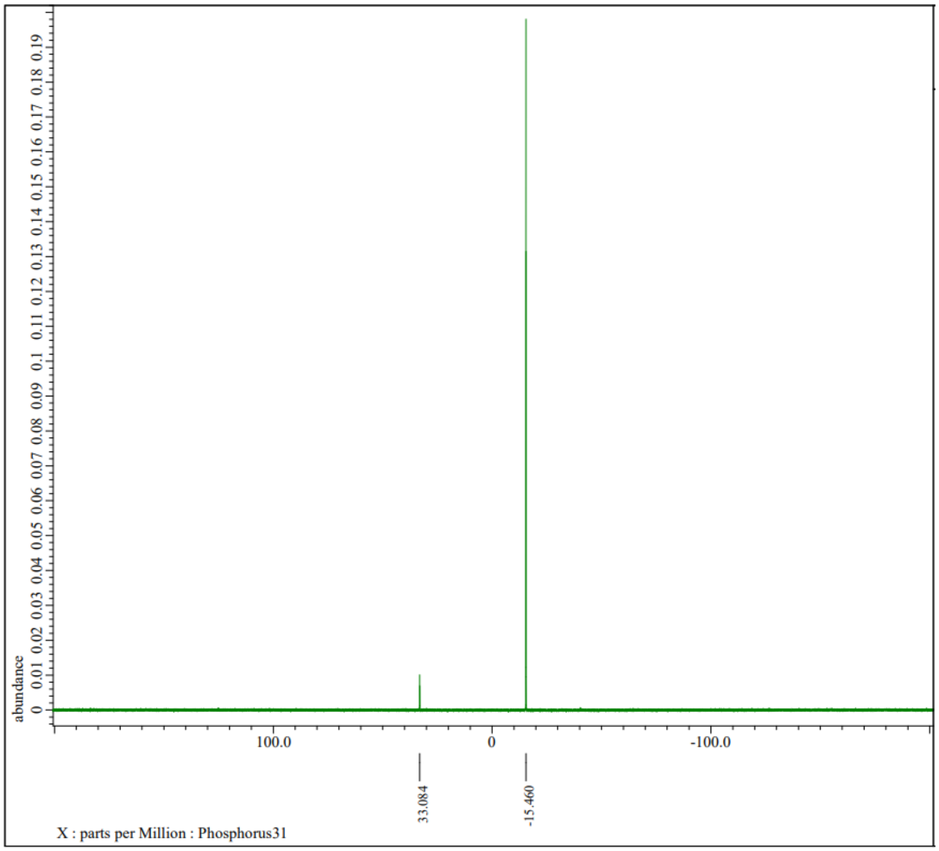

Figure S4. ^{1}H-NMR, ^{13}C-NMR and ^{31}P-NMR spectra of (**5**) obtained by method A.

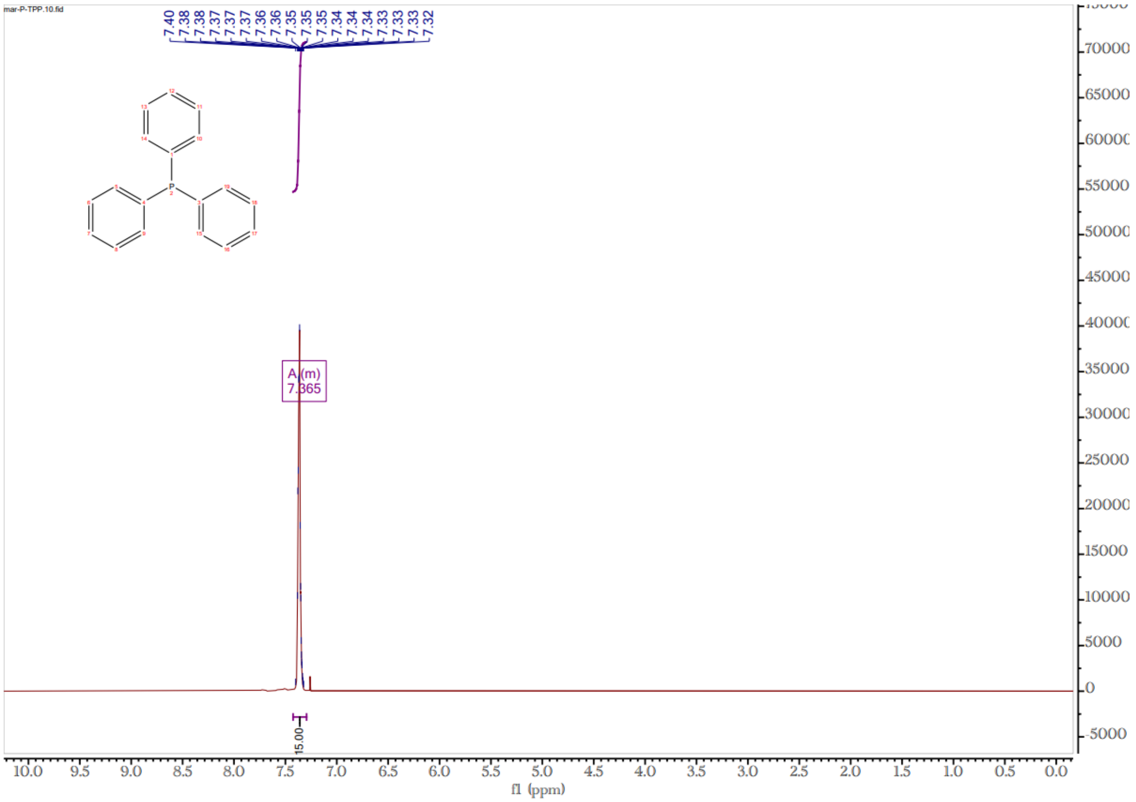
mar-P-TPP.10.fid
7.40
7.38
7.38
7.37
7.37
7.37
7.36
7.36
7.35
7.35
7.35
7.34
7.34
7.34
7.33
7.33
7.33
7.33
7.32
A (m)
7.365
15.00
f1 (ppm)

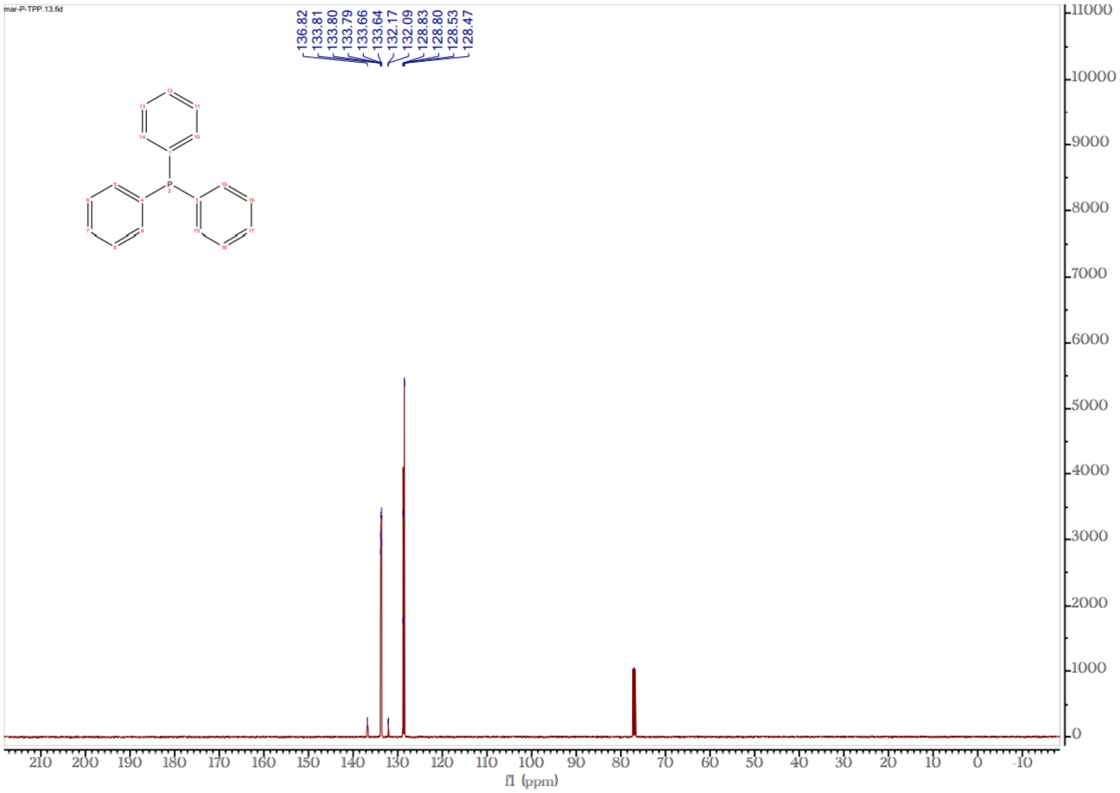
mar-P-TPP.13.fid
136.82
133.81
133.80
133.79
133.66
133.64
132.17
132.09
128.83
128.80
128.53
128.47
f1 (ppm)

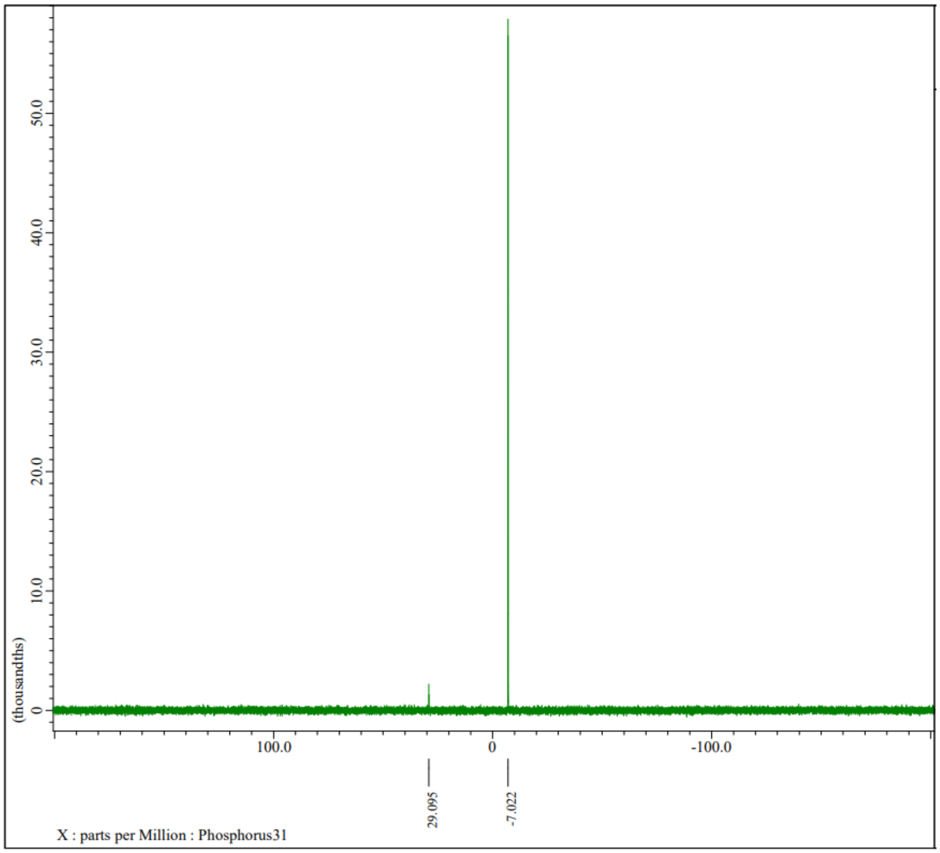

Figure S5. ^{1}H-NMR, ^{13}C-NMR and ^{31}P-NMR spectra of **(6)** obtained by method A.

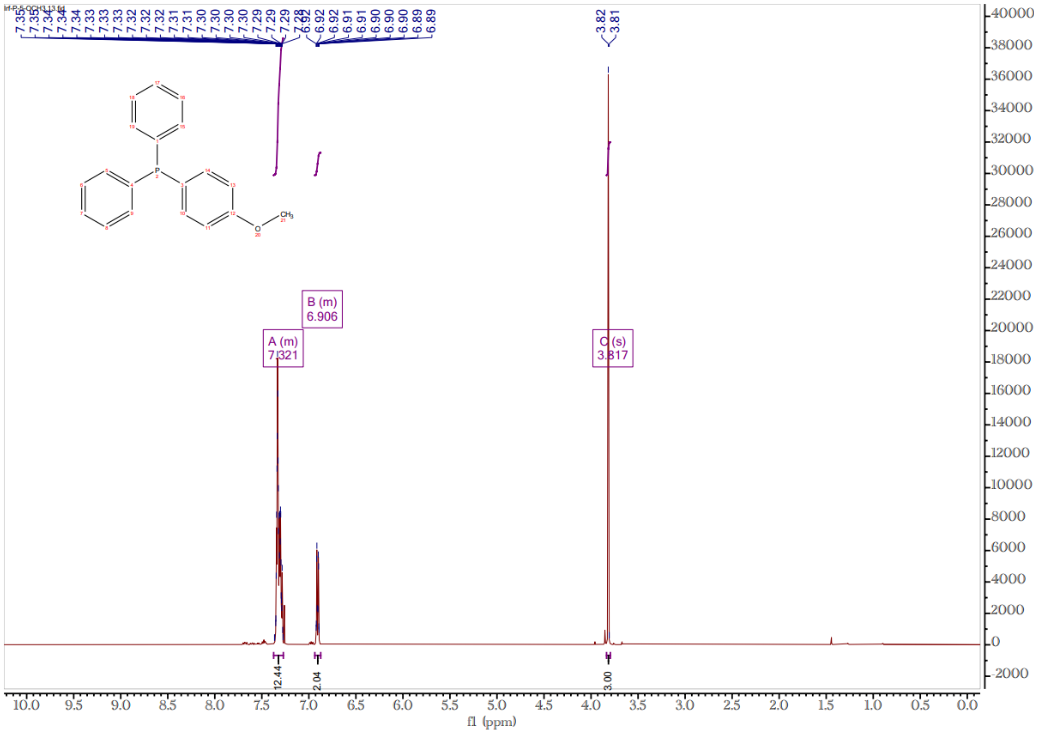

lrl-P-5-OCH3.13.fid
A (m)
7.321
B (m)
6.906
C (s)
3.817
12.44
2.04
3.00
f1 (ppm)

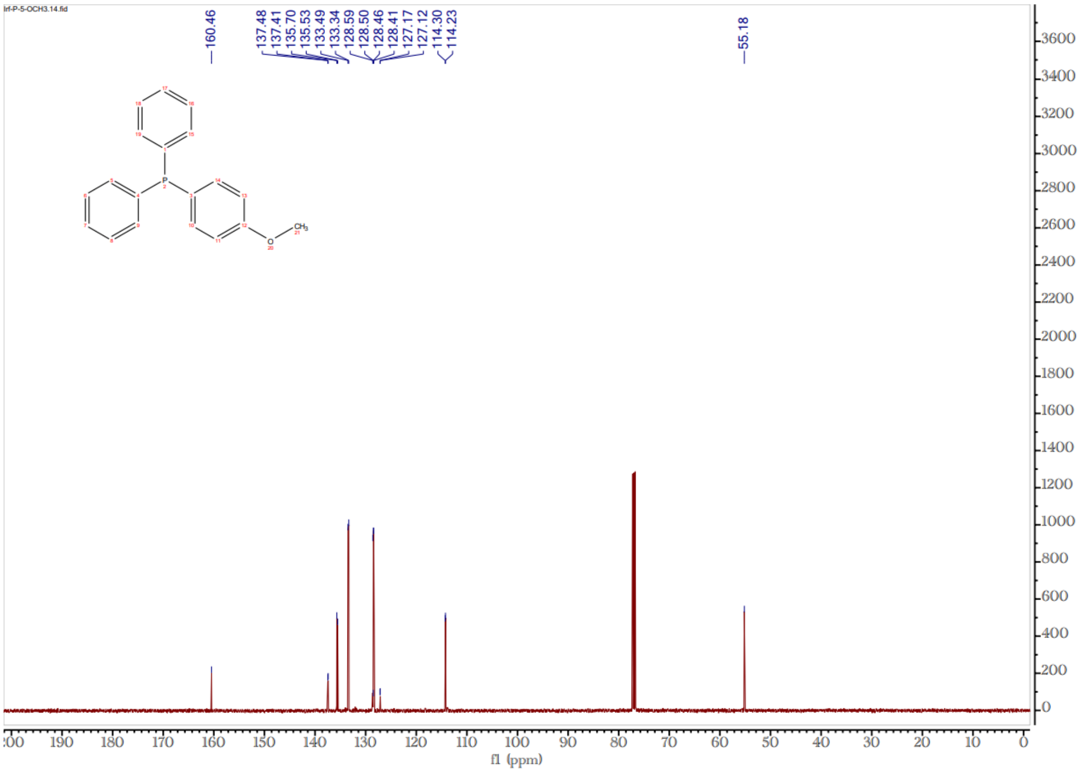

lrl-P-5-OCH3.14.fid
160.46
137.48
137.41
135.70
135.53
133.49
133.34
128.59
128.50
128.46
128.41
127.17
127.12
114.30
114.23
55.18
f1 (ppm)

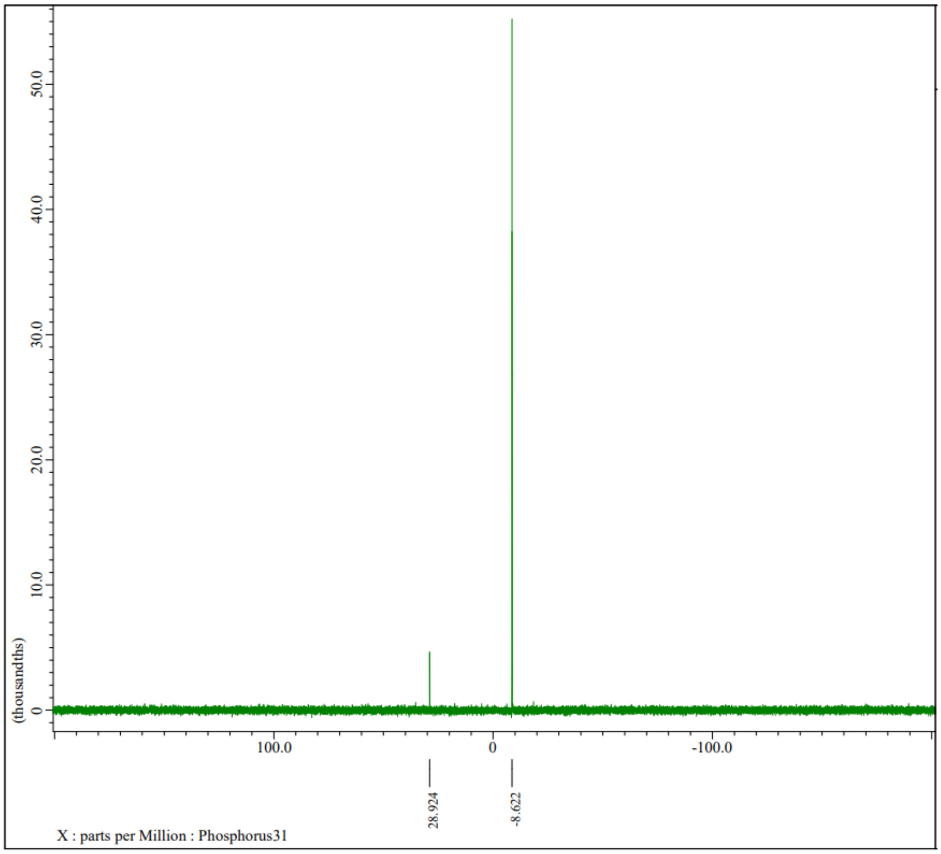

Figure S6. ^{1}H-NMR, ^{13}C-NMR and ^{31}P-NMR spectra of **(7)** obtained by method A.

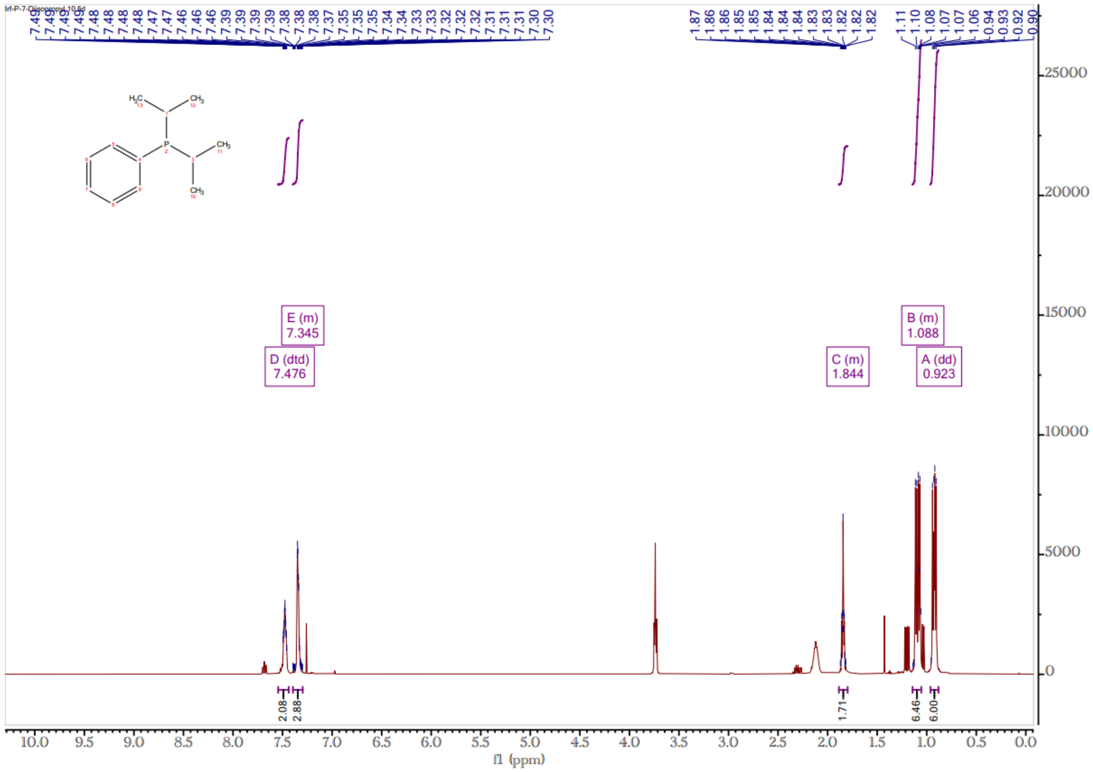

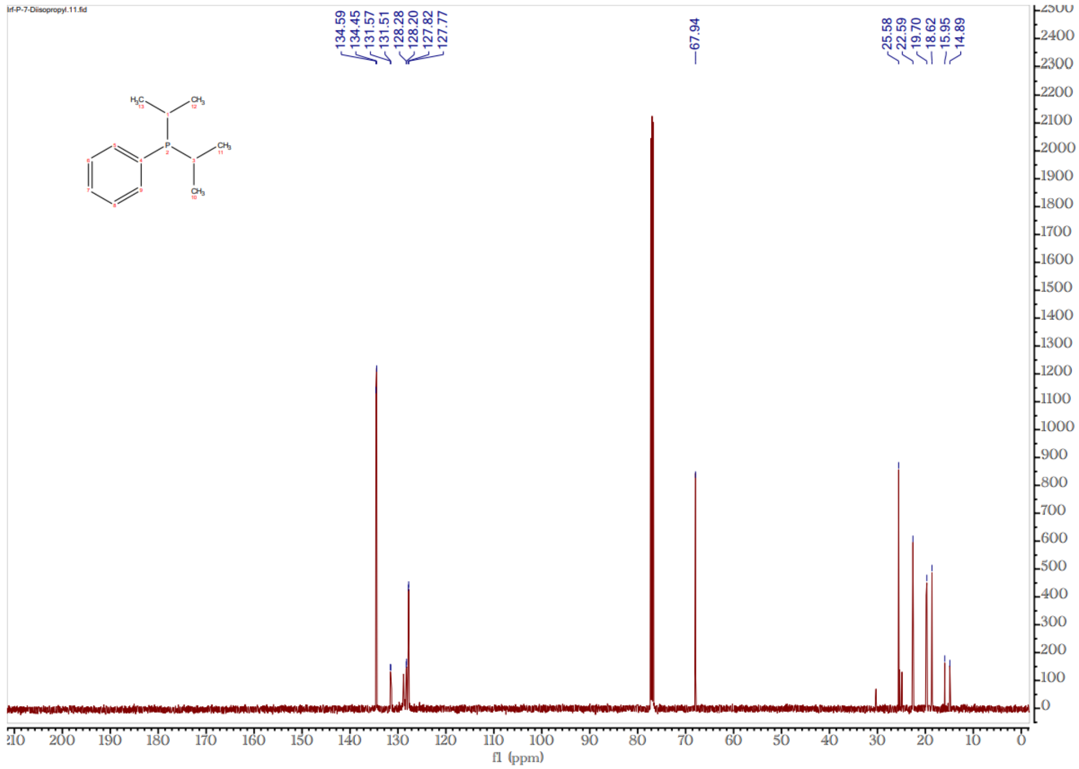

Figure S7. ^{1}H-NMR, ^{13}C-NMR and ^{31}P-NMR spectra of (**9**) obtained by method B.

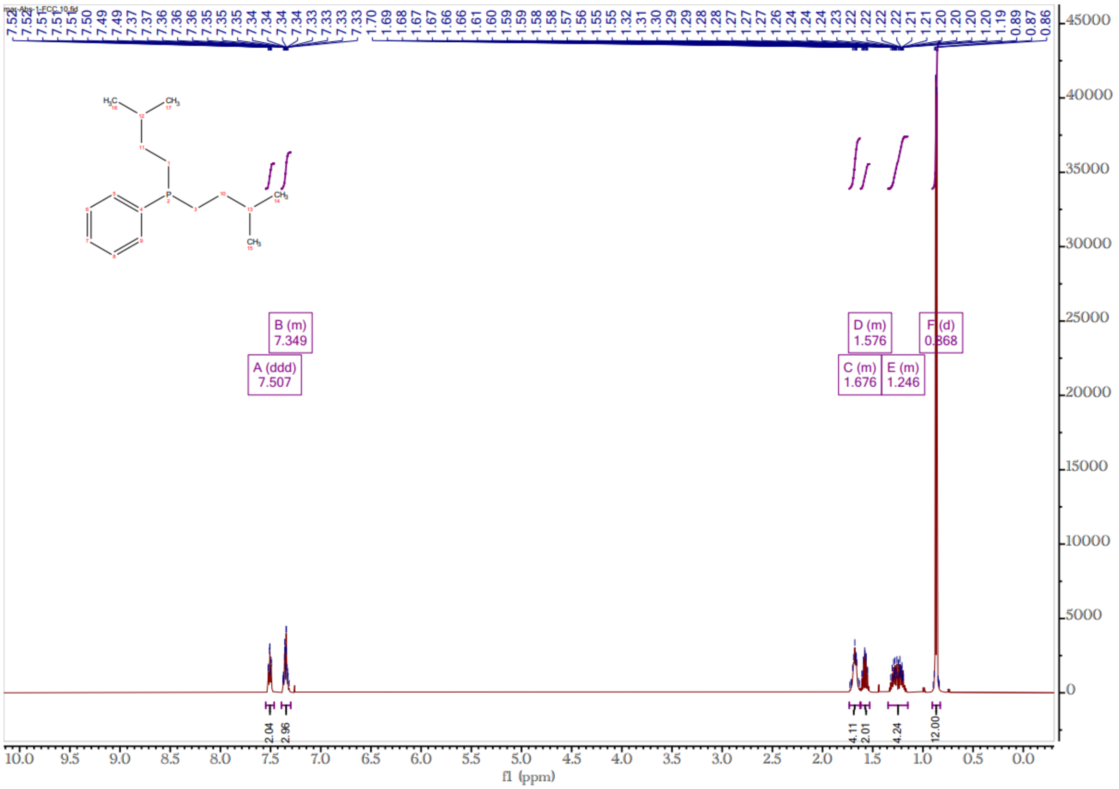
mar-Ahs-1-FCC.10.fid
7.52 7.52 7.51 7.51 7.50 7.50 7.49 7.37 7.37 7.36 7.36 7.35 7.35 7.35 7.34 7.34 7.34 7.33 7.33 7.33
1.70 1.69 1.68 1.67 1.67 1.66 1.66 1.61 1.60 1.59 1.59 1.58 1.58 1.57 1.56 1.55 1.55 1.32 1.31 1.30 1.29 1.28 1.28 1.27 1.27 1.26 1.24 1.24 1.23 1.22 1.22 1.22 1.22 1.21 1.20 1.20 1.20 1.19 0.89 0.87 0.86
B (m) 7.349
A (ddd) 7.507
D (m) 1.576
C (m) 1.676
E (m) 1.246
F (d) 0.868
2.04
2.96
4.11
2.01
4.24
12.00
f1 (ppm)

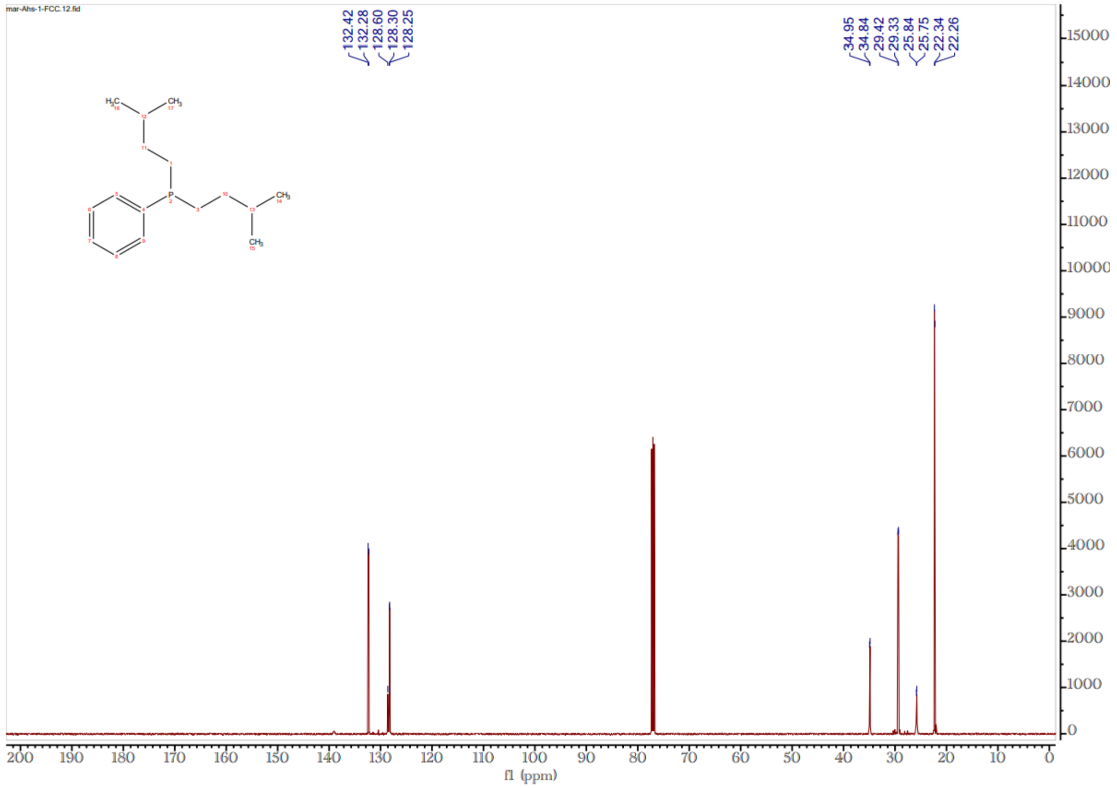
mar-Ahs-1-FCC.12.fid
132.42 132.28 128.60 128.30 128.25
34.95 34.84 29.42 29.33 25.84 25.75 22.34 22.26
f1 (ppm)

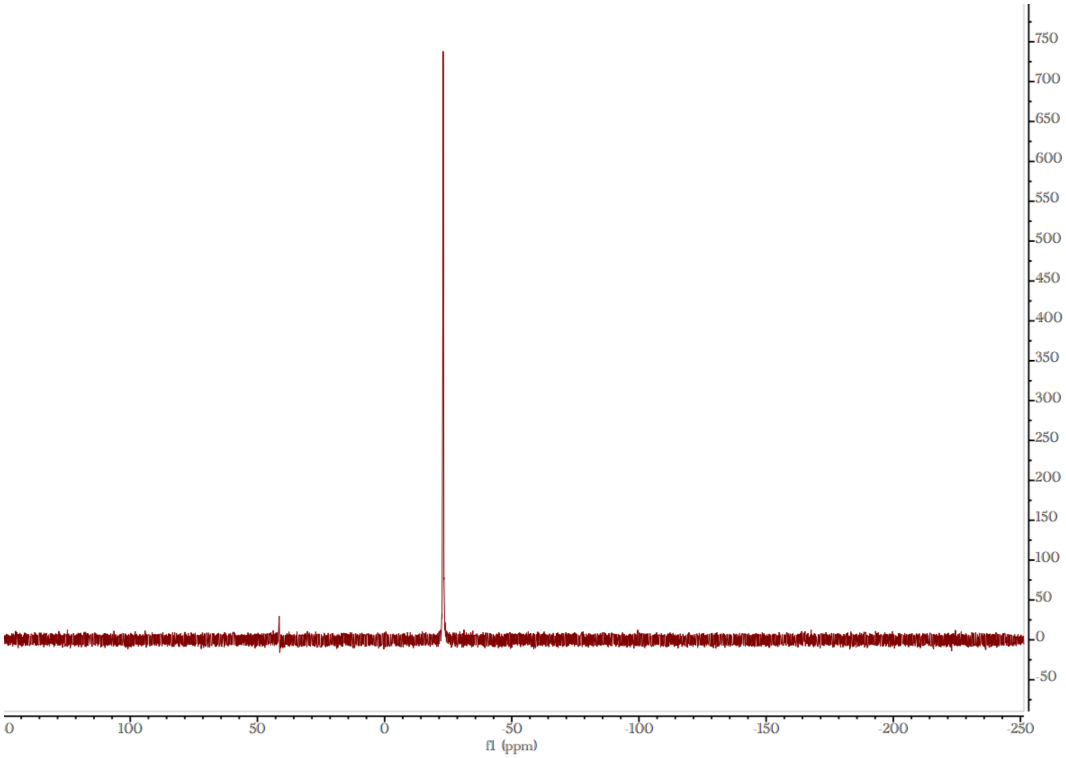

Figure S8. ¹H-NMR, ¹³C-NMR, ³¹P-NMR spectra of (**10**) obtained by method B.

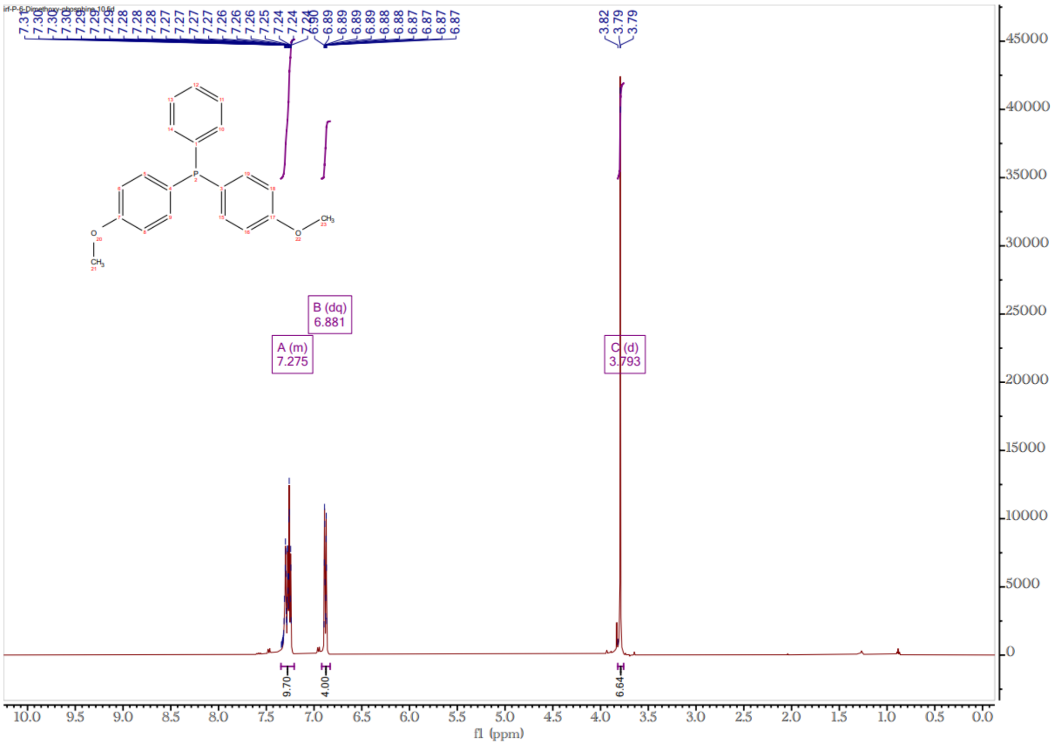

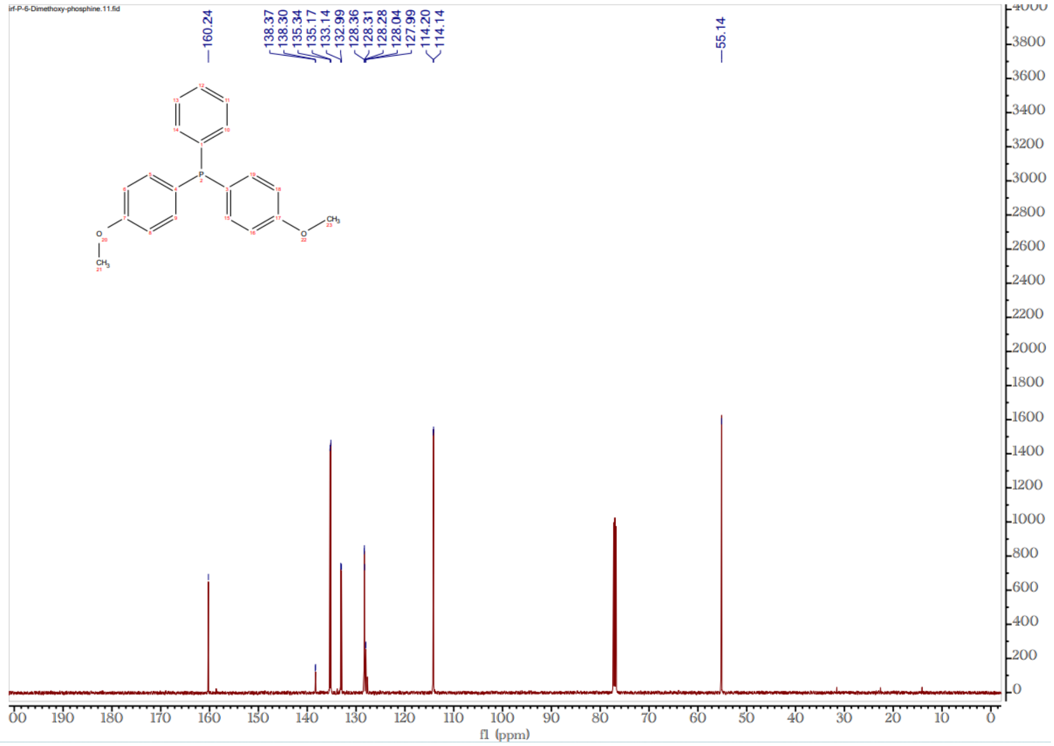

Figure S9. ^{1}H-NMR and ^{13}C-NMR spectra of **(11)** obtained by method B.

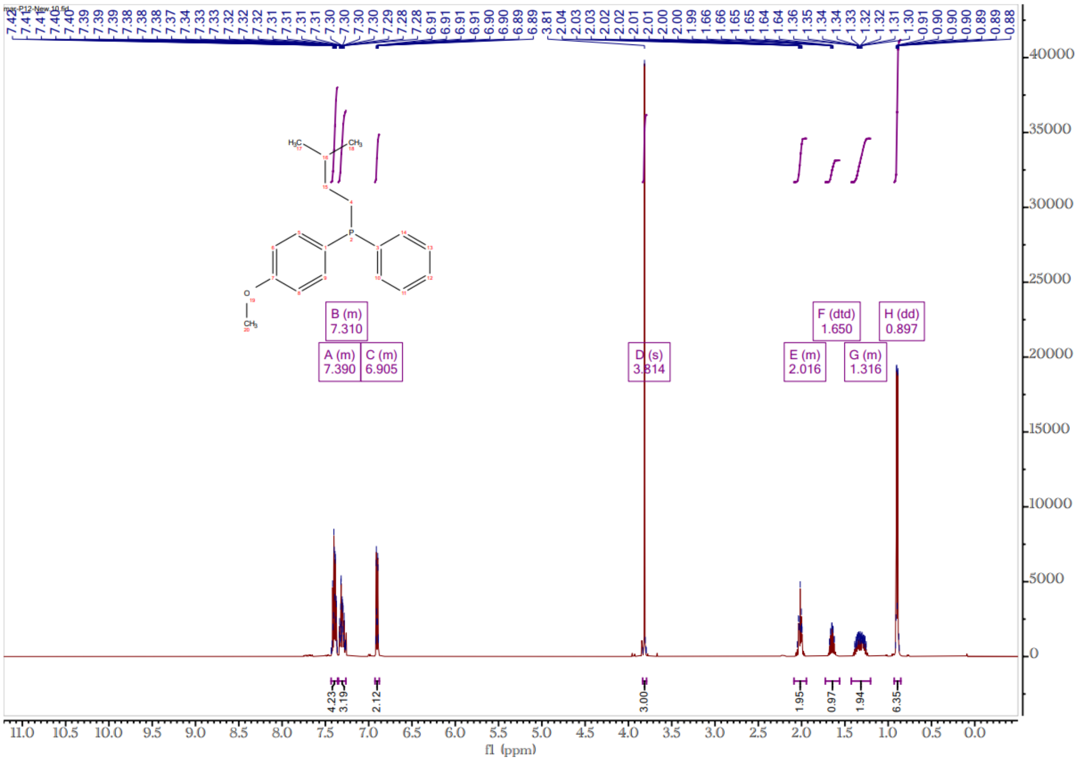
mar-P12-New.10.fid
A (m) 7.390
B (m) 7.310
C (m) 6.905
D (s) 3.814
E (m) 2.016
F (dtd) 1.650
G (m) 1.316
H (dd) 0.897
4.23
3.19
2.12
3.00
1.95
0.97
1.94
6.35
f1 (ppm)

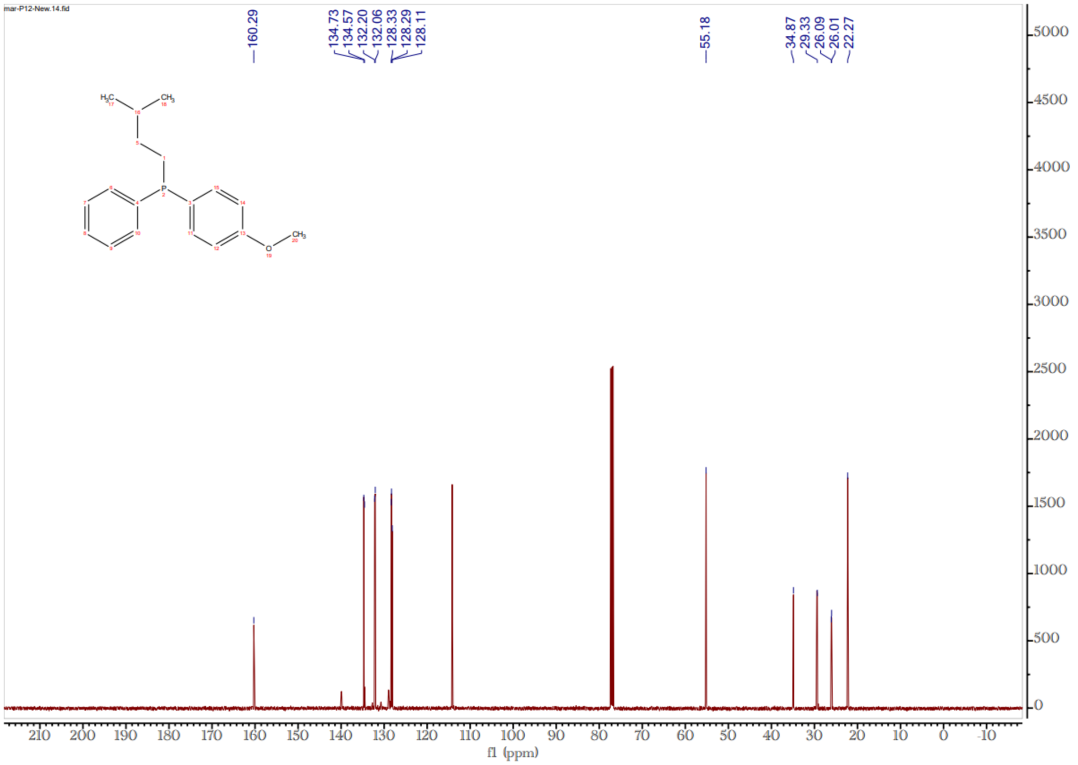
mar-P12-New.14.fid
160.29
134.73
134.57
132.20
132.06
128.33
128.29
128.11
55.18
34.87
29.33
26.09
26.01
22.27
f1 (ppm)

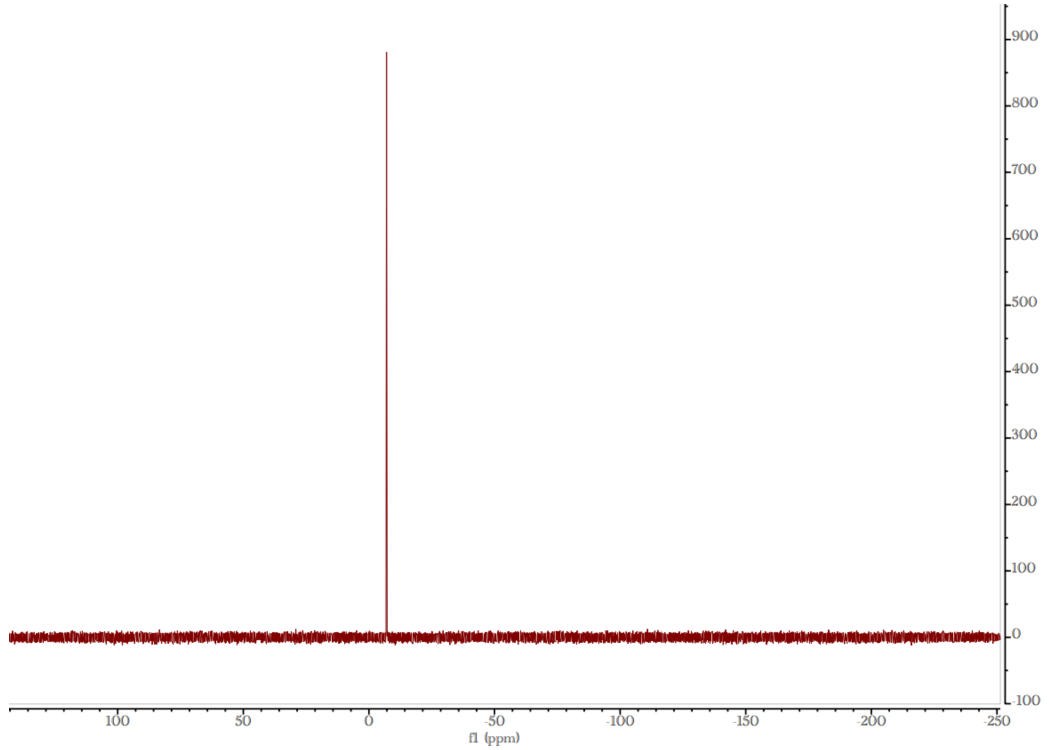

Figure S10. ^{1}H-NMR, ^{13}C-NMR and ^{31}P-NMR of **(12)** obtained by method C.

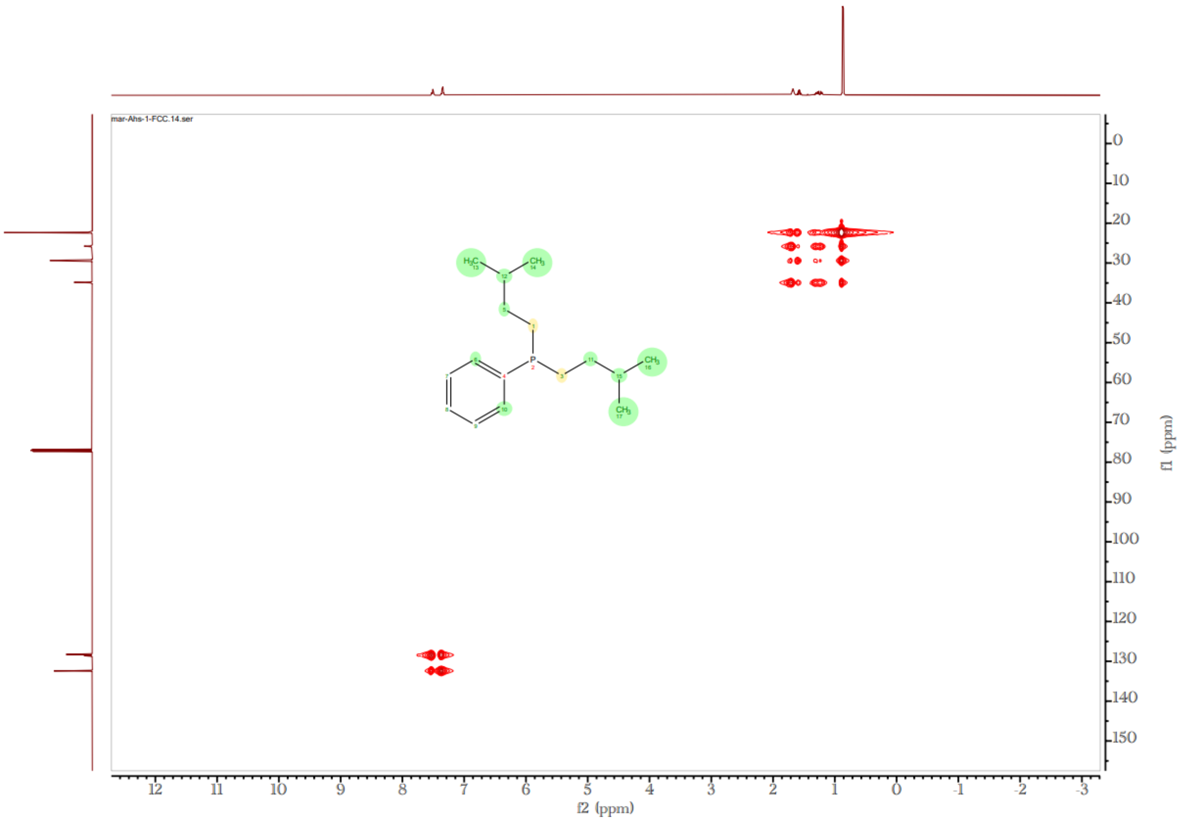

Figure S11. HMBC spectrum of compound **(10)** obtained by method B.

Table T1: Cartesian coordinates for the optimized structure of 5.

0 1

P	-0.27315200	-0.18579600	-0.58422600
C	-0.08598400	1.57986400	-0.22324800
C	1.05562400	2.30459700	-0.61843900
C	-1.17449500	2.26709600	0.34481300
C	1.11612200	3.67778800	-0.41189500
H	1.88415200	1.80320400	-1.10052000
C	-1.09554200	3.63724200	0.56693100
H	-2.06777400	1.71837500	0.61537000
C	0.04662300	4.34421000	0.18791900
H	1.99444700	4.23015300	-0.72438800
H	-1.92870900	4.15578400	1.02622000
H	0.09864800	5.41469700	0.34849900
C	-1.81591400	-0.87654200	-0.22233000
C	-2.72231300	-1.28300900	-1.28066100
C	-2.27415100	-1.05376200	1.15382900
C	-3.95233600	-1.81232100	-0.98470500
H	-2.42156600	-1.16845700	-2.31671100
C	-3.51916400	-1.58379300	1.40145400
H	-1.63402700	-0.77006500	1.98173700
C	-4.39189700	-1.97874200	0.36040800
H	-4.60376300	-2.10993500	-1.80127700
H	-3.83288400	-1.70529700	2.43507100
H	-5.36613000	-2.39486400	0.57957600
C	1.14997800	-1.18270800	0.06026200
H	1.02785500	-2.14207900	-0.44871500
H	0.89359700	-1.36052200	1.11368900
C	2.58367100	-0.65685000	-0.06483300
H	2.67063500	0.28879200	0.47643900
H	2.81787800	-0.44848100	-1.11544600
C	3.64226700	-1.63286900	0.49418700
H	3.34699900	-1.88894600	1.52031800
C	3.73298500	-2.93361600	-0.31569100
H	2.79177200	-3.48879900	-0.32002300
H	4.00531000	-2.72387100	-1.35592700
H	4.49802300	-3.59403000	0.10168000
C	5.01054300	-0.93958100	0.55774100
H	4.97167700	-0.03563800	1.17236900

H	5.76677100	-1.60418900	0.98456600
H	5.34827300	-0.65091700	-0.44358900

Table T2: Cartesian coordinates for the optimized structure of 10

0 1

P	0.07333500	-0.36561600	-0.39955600
C	-1.15961400	-1.54639200	-0.13199000
C	-1.88256500	-2.14281600	-1.24186400
C	-1.54354800	-1.96089200	1.21605100
C	-2.87250600	-3.06511000	-1.01731700
H	-1.63407400	-1.85795700	-2.25876000
C	-2.54269100	-2.88825700	1.39055800
H	-1.04167400	-1.54060100	2.08086800
C	-3.23422500	-3.47020400	0.29942700
H	-3.39016600	-3.49438100	-1.87046100
H	-2.80466900	-3.18049500	2.40428100
H	-4.01792800	-4.19795600	0.46229800
C	1.77499800	-0.85422500	0.11885300
H	1.98601900	-1.75881100	-0.45688000
H	1.68771200	-1.17086800	1.16694300
C	2.87822000	0.20008000	-0.04792200
H	2.64023100	1.07271600	0.56728200
H	2.90729700	0.54632000	-1.08817200
C	4.28255300	-0.30234100	0.35011200
H	4.21197400	-0.71022700	1.36722800
C	-0.24398900	1.35528900	0.19172000
H	-0.21835600	1.30788900	1.28940100
H	0.59949300	1.97300300	-0.12466000
C	-1.58526900	1.91919200	-0.29482300
H	-2.37618200	1.20245000	-0.05508500
H	-1.56796900	2.01030200	-1.38763200
C	-1.94981900	3.28569700	0.31880100
H	-1.91829700	3.17562800	1.41116200
C	5.27050700	0.87220100	0.38161500
H	4.94156700	1.65201500	1.07454900
H	6.26389200	0.54036500	0.69600800
H	5.36949200	1.32484800	-0.61099900
C	4.79486800	-1.41439200	-0.57560600
H	4.14893400	-2.29571000	-0.56530600
H	4.86160700	-1.05762400	-1.60930800

H	5.79346000	-1.73925100	-0.27079500
C	-3.38273800	3.67151800	-0.07354000
H	-4.10078200	2.90884400	0.24080900
H	-3.67403000	4.61934000	0.38772800
H	-3.47135800	3.78827400	-1.15912100
C	-0.96307100	4.39326100	-0.07665200
H	0.05513200	4.18022600	0.25886100
H	-0.93919300	4.52038700	-1.16467200
H	-1.25881500	5.34985700	0.36324400

Table T3: Cartesian coordinates for the optimized structure of PBu_3

0 1

P	-0.30308200	-0.44771100	0.61804900
C	-1.86259500	-1.06590800	-0.21664000
H	-1.91766200	-2.14017200	-0.00856600
H	-1.76457300	-0.95704800	-1.30368500
C	-3.14654900	-0.38488300	0.27474400
H	-3.08657300	0.69711300	0.10533200
H	-3.23682100	-0.51903300	1.35927100
C	-0.01675600	1.12829800	-0.36063800
H	0.31570900	0.86852400	-1.37300500
H	-0.99606700	1.60797800	-0.46441600
C	0.95636200	2.12170900	0.28977900
H	1.93776200	1.65408000	0.42814900
H	0.59661500	2.37373300	1.29446100
C	-5.69447500	-0.24747500	0.08722400
H	-5.67026900	0.83105900	-0.09810100
H	-6.57700000	-0.65152800	-0.41645000
H	-5.82840200	-0.39545500	1.16341100
C	2.08941700	4.41021000	0.13003300
H	3.08754000	3.97743000	0.25085100
H	2.19120600	5.31749200	-0.47189100
H	1.73521400	4.70690900	1.12227100
C	0.91446800	-1.62898900	-0.17734000
H	0.80648100	-1.58325000	-1.26797900
H	0.59876200	-2.63220200	0.13195800
C	2.38106500	-1.41656900	0.21979500
H	2.72260600	-0.43401600	-0.12485200
H	2.46775500	-1.40591100	1.31308200
C	3.31658900	-2.49204200	-0.34904300

H	3.22505300	-2.50783900	-1.44170100
H	2.98407900	-3.47701200	-0.00001000
C	4.78294400	-2.27979200	0.03839400
H	5.42257100	-3.06241100	-0.37894400
H	5.15306000	-1.31706700	-0.32810900
H	4.90951500	-2.29093900	1.12554200
C	-4.41175900	-0.92209400	-0.40767900
H	-4.47829400	-2.00369300	-0.23952600
H	-4.32136100	-0.78623500	-1.49203600
C	1.12949000	3.41145100	-0.52313000
H	0.14884900	3.88266500	-0.66163600
H	1.49127600	3.15898700	-1.52715200
